# Phylogenetic relationships and phylogeography of relevant lineages within the complex Campanulaceae family in Macaronesia

**DOI:** 10.1002/ece3.3640

**Published:** 2017-11-23

**Authors:** Tiago Menezes, Maria M. Romeiras, Miguel M. de Sequeira, Mónica Moura

**Affiliations:** ^1^ CIBIO Research Centre in Biodiversity and Genetic Resources InBIO Associate Laboratory Faculdade de Ciências e Tecnologia Universidade dos Açores Ponta Delgada Azores Portugal; ^2^ Linking Landscape, Environment, Agriculture and Food (LEAF) Instituto Superior de Agronomia Universidade de Lisboa Lisbon Portugal; ^3^ Centre for Ecology, Evolution and Environmental Changes (cE3c) Faculdade de Ciências Universidade de Lisboa Lisbon Portugal; ^4^ Madeira Botanical Group Faculdade de Ciências da Vida Universidade de Madeira Alto da Penteada Funchal Madeira Portugal

**Keywords:** Campanulaceae, divergence time estimation, endemic, hybridization, Macaronesia, phylogeny

## Abstract

Macaronesia has long been recognized as a natural model for studying evolutionary processes in plant diversification. Several studies have attempted to focus on single lineages, and few have covered the diversification of a family across all the archipelagos. We used a comprehensive sample to clarify the phylogenetic relationships and the biogeographic history of the Macaronesian Campanulaceae. Hypotheses related to the colonization of these archipelagos will be used to examine the diversification patterns of different lineages. We sequenced the ITS region and six cpDNA markers (*atpB*,* matK*,* petD*,* rbcL*,* trnL‐F*, and *psbA‐trnH*) from 10 Campanulaceae species, including seven endemic species in Macaronesia. The phylogeny of these taxa was reconstructed using maximum parsimony, maximum likelihood, and Bayesian inference. To study the relationships within each lineage, haplotype networks were calculated using NeighborNet and TCS algorithms. Moreover, data were combined with fossil information to construct time‐calibrated trees for the Macaronesian Campanulaceae species. The phylogenetic analyses are largely congruent with current taxon circumscriptions, and all the endemic genera formed monophyletic clades, namely *Azorina* in Azores; *Musschia* in Madeira; and *Campanula* in Cape Verde. The *Azorina* clade and the Cape Verde endemic *Campanula* may share a common ancestor in North Africa, and the divergence was dated ca. 12.3 million years ago (Mya). The divergence of the *Musschia* clade began in the Pliocene ca. 3.4 Mya. Moreover, several examples of intraspecific variation were revealed among the native species with a clear geographic structured patterns, suggesting that cryptic diversity might exist within the native Macaronesian Campanulaceae when compared to the close mainland taxa (e.g., *Campanula erinus*,* Trachelium caeruleum*), but additional studies are needed to support the molecular data. This study highlights the power of combining data (e.g., phylogeny and divergence times, with species distribution data) for testing diversification hypotheses within the unique Macaronesian flora, providing useful information for future conservation efforts.

## 1 INTRODUCTION

Islands are biologically simpler than continents and consequently provide perfect geographic and historical settings for the study of species colonization and diversification (Parent, Caccone, & Patren, 2008). The Macaronesian archipelagos (i.e., Azores; Madeira incl. Selvages; Canaries and Cape Verde) occupy a unique position in the history of evolutionary studies, due to its importance as a past and present conceptual landmarks (Darwin, 1859; Mayr, 1967; Wallace, 1880) and because it continues to shape our understanding of evolutionary biology (Harter et al., 2015; Johnson, Price, Price, & Stacy, 2015; Stacy, Johansen, Sakishima, Price, & Pillon, 2014).

Macaronesia displays a high degree of plant endemicity (Carine, Santos Guerra, Guma, & Reyes‐Betancort, 2010; Caujape‐Castells et al., 2010; Cosner, Raubeson, & Jansen, 2004), related to its geographic location (and variable isolation), geological origin, and climatic history (Jardim & Menezes de Sequeira, 2008). Although most of the phylogenetic studies have been focused on single lineages with emphasis on Canaries, which displays ca. one‐third of the endemic plant diversity of Macaronesia, some studies include two or more Macaronesian archipelagos (Carine, Francisco‐Ortega, Santos‐Guerra, & Russell, 2004; Mort et al., 2015; Moura, Carine, Malékot, et al., 2015; Moura, Carine, & Sequeira, 2015; Romeiras et al., 2011). Nonetheless, broad‐scale studies focused on a plant family covering native and endemics and comparing distinct patterns of phylogenetic structure among the five archipelagos was not yet addressed for this hotspot region. To compare phylogenetic and distribution patterns within all the archipelagos, this study is focused on the Campanulaceae Juss. family, which is characterized by a great number of native species, including some endemic genera in Macaronesia.

The family Campanulaceae includes 88 genera and ca. 2,385 species (The Plant List, 2017). Since De Candolle (1839) and Bentham and Hooker (1876), different circumscriptions of the Campanulaceae have been proposed, for example, by Gustafsson and Bremer (1995) who consider the Campanulaceae clade as five distinct families, while others, such as Lammers (1998) and Brummitt (2007), treat the five families as subfamilies, namely (1) Lobelioideae Burnett, the largest subfamily, comprising ca. 1,200 species, half of which are native to South America; (2) Campanuloideae Burnett, with ca. 1,000 species distributed worldwide, with a center of diversity in the Holarctic, including the Macaronesian Islands; (3) Cyphioideae (A. DC.) Walp. with ca. 65 perennial herbs restricted to Africa; (4) Nemacladoideae Lammers with ca. 19 species mainly distributed in the south‐western USA and northern Mexico; (5) and Cyphocarpoideae Miers., which includes only three annual species endemics in the Atacama Desert of Chile (Crowl et al., 2016)].

**Figure 1:**
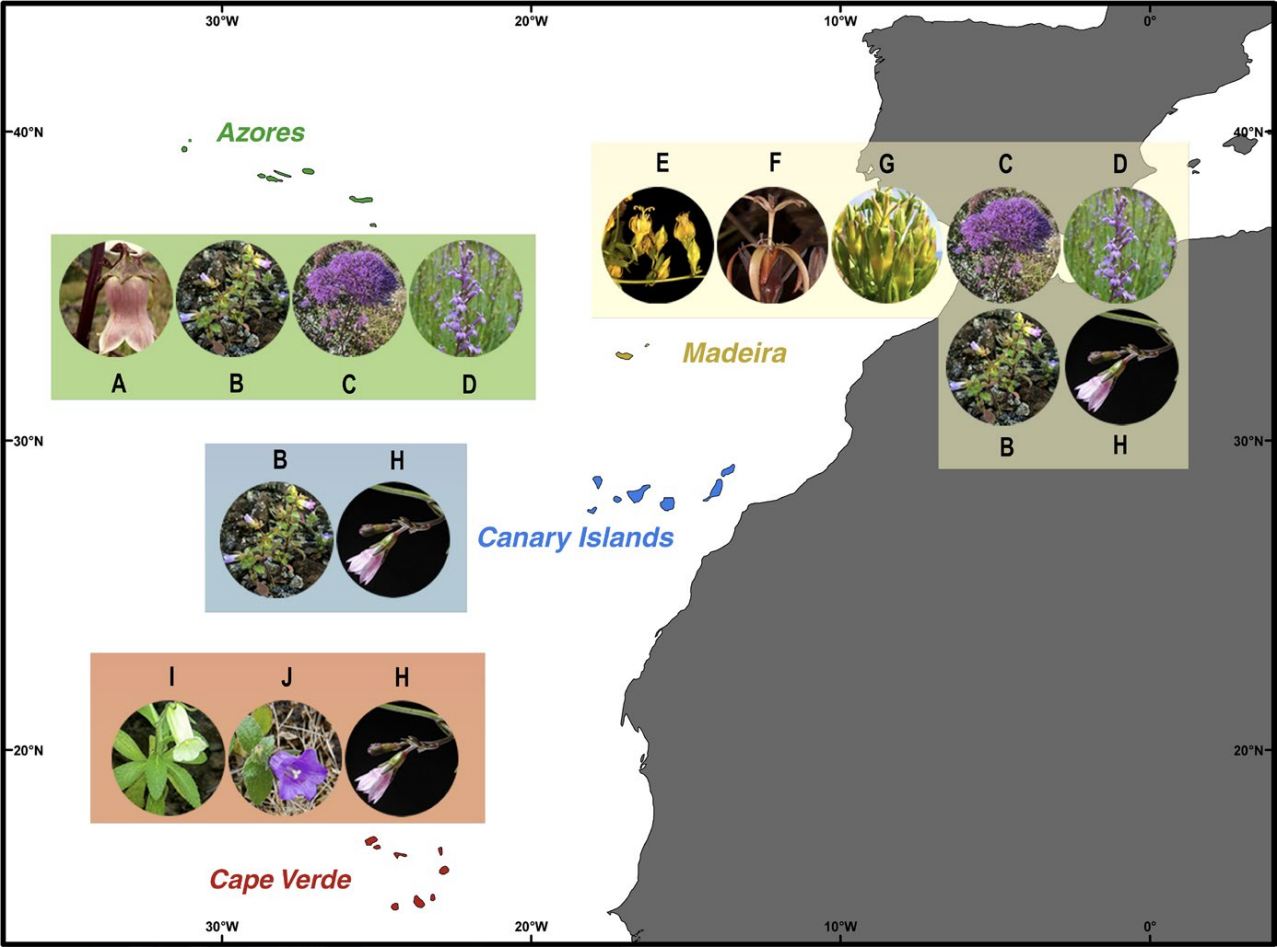
Distribution of the target species in Macaronesia. Plants pictures are as follows: (A) *Azorina vidalii*; (B) *Campanula erinus*; (C) *Trachelium caeruleum*; (D) *Lobelia urens*; (E) *Musschia aurea*; (F) *M. wollastonii*; (G) *M. isambertoi*; (H) *Wahlenbergia lobelioides* subsp. *lobelioides*; (I) *C. bravensis*; (J) *C. jacobaea*

In Macaronesia, Campanulaceae includes two endemic genera and nine (or eleven) endemic species are currently recognized. *Azorina* Feer is a monospecific genus (*Azorina vidalii* [H.C. Watson] Feer) endemic from the Azores and *Musschia* Dumort. is endemic from the Madeira archipelago, with three recognized species: *M. aurea* (L.f.) Dumort.; *M. isambertoi* M.Seq., R. Jardim, M. Silva & L. Carvalho; and *M. wollastonii* Lowe (Menezes de Sequeira, Espírito‐Santo, Aguiar, Capelo, & Honrado, 2012). In the Canary archipelago, two endemic species are currently recognized: *Canarina canariensis* (L.) Vatke and *Campanula occidentalis* Y.Nymann. In Cape Verde, two endemics, *Campanula bravensis* (Bolle) A.Chev. and *Campanula jacobaea* C.Sm. ex Hook, were listed for the archipelago (Sánchez‐Pinto et al., 2005), and recently, Gardère (2015) described two new species for Santo Antão Island (i.e., *C. feijoana* Gardère and *C. hortelensis* Gardère, which were previously identified as *C. jacobaea*). Finally, *Wahlenbergia lobelioides* (L.f.) Link subsp. *lobelioides* is endemic in three Macaronesian archipelagos: Madeira (Menezes de Sequeira et al., 2012), Canaries (Ginovés et al., 2010), and Cape Verde (Sánchez‐Pinto et al., 2005). Moreover, in Macaronesia there are several other native (although nonendemic), and non‐native (fully naturalized or casual species) with uncertain status (Ginovés et al., 2010; Menezes de Sequeira et al., 2012; Sánchez‐Pinto et al., 2005; Silva, Moura, Schaefer, Rumsey, & Dias, 2010), requiring a more detailed taxonomic and molecular studies in this region, as the case of *Campanula erinus* L., *Lobelia urens* L. and *Trachelium caeruleum* L. (see Figure 1), the last one with doubtful status for the Azores archipelago (Silva et al., 2010).

Phylogenetic relationships within Campanulaceae remain highly controversial, and a complex biogeographic history has been recently reported in several studies (e.g., Cosner et al., 2004; Crowl et al., 2014; Eddie, Shulkina, Gaskin, Haberle, & Jansen, 2003; Haberle et al., 2009; Roquet et al., 2008). One of the most comprehensive studies on the Campanulaceae family was presented by Crowl et al. (2016), providing a broad phylogenetic and phylogeographic perspective which included chromosomal and morphological data. Previously, Crowl et al. (2014) had produced a first phylogenetic analysis conjointly applying several molecular markers used in former studies within the subfamily Campanuloideae, namely the chloroplast markers *atpB*,* matK*,* petD*,* rbcL*, and *trnL‐F* and the nuclear region ITS. However, the authors concluded that ITS was inefficient on Campanuloideae due to difficulties on the alignment of sequences, high levels of homoplasy, and concerted evolution, although it provided information at the species level. Alternatively, two single‐copy nuclear loci from the PPR genes family (pentatricopeptide repeat: PPR11 and PPR70) provided independent estimations of relationships, uncovering hybridization events.

To date, only some of the native Macaronesian Campanulaceae species have been included in these phylogenetic studies, and the relationships among some of the genera were not yet clarified. For instance, paraphyly and polyphyly were found between *Campanula* L. and *Wahlenbergia* Schrad. ex Roth and conflicts between morphological and molecular data were reported for Azorean endemic *Azorina*, which was placed inside the *Campanula* clade in recent studies (Crowl et al., 2014; Olesen, Alarcón, Ehlers, Aldasoro, & Roquet, 2012; Roquet et al., 2008, 2009).

The biogeographic history in this family, namely in *Campanula*, is complex and involves a considerable number of migrations (e.g., from the Balkans to western Asia) being particularly critical in its diversification due to orogenic activity that took place in this region during the Late Neogene, and which could have promoted isolation and allopatric speciation within/among lineages, followed by range expansion and posterior isolation to give rise to new endemics, as well as several long‐distance (independent) dispersal events to Macaronesia (Roquet et al., 2009).

Some phytogeographic studies were recently published by Mairal, Pokorny, Aldasoro, Alarcón, and Sanmartín (2015), Mairal, Sanmartín, et al. (2015), who suggested that for *C. canariensis*, which is widespread across several Canary Islands, the ocean is apparently less of a barrier than topographic relief within volcanic islands; and the paleo‐islands of Tenerife have probably acted as both genetic refuge and sources of new diversity within and between islands. Moreover, Alarcón, Roquet, García‐Fernández, Vargas, and Aldasoro (2013) sampling the Cape Verdean *Campanula* species pointed to a recent divergence (1.0 million years ago [Mya]) of this clade from its sister species *C. balfourii* J. Wagner & Vierh., which is endemic in Socotra Island. A recent study conducted by García‐Aloy et al. (2017) proposed the Northwest Africa as a hub of diversification in Mediterranean plants and their biogeographic analyses suggested that the most basal divergence events within *Azorina* group involved northwest Africa as the ancestral area, and date back to the Late Miocene‐Pliocene (ca. 8.7–13.3 Mya).

There is still, however, an important gap related to the phylogenetic knowledge of the Macaronesian lineages, namely related to the endemic *Musschia* species in Madeira archipelago, where only a few individuals of *M. aurea* and *M. wollastonii* have been included in molecular studies, leaving out *M. isambertoi*. Indeed, most of the studies in Macaronesian Campanulaceae have used sparse sampling, thus aspects related to the diversification and colonization patterns of particular groups remain to be further clarified, such as the estimation of divergence times for several endemic lineages. Finally, the integration of both phylogenetic relationships and distribution data will constitute important information for conservation actions.

This study aimed to (1) determine the phylogenetic relationships between native and endemic Campanulaceae species occurring in the Azores, Madeira, Canaries, and Cape Verde, as well as their relationships with continental taxa; (2) determine the potential processes driving diversification in Campanulaceae within the Macaronesian region with divergence time estimations; and (3) contribute with genetic data to assist in future conservation plans.

## 2 METHODS

### 2.1 Study area

The study area includes all the Macaronesian archipelagos, namely the Azores archipelago with nine islands and some islets; Madeira archipelago comprising Madeira, Porto Santo, and the Desert as subarchipelago composed of three small islands; the Canary archipelago including seven main islands and six islets; and finally Cape Verde, the southernmost islands of Macaronesia, with 10 islands. These archipelagos are characterized by high mountain ranges with a great habitat diversity, having a huge endemic plant diversity within the Mediterranean Basin Biodiversity Hotspot. For a description of all Macaronesian archipelagos and their biodiversity, see Vanderpoorten et al. (2011).

### 2.2 Sampling

Leaf samples of 46 individuals were collected in natural populations, preserved in silica dried and stored in vacuum‐sealed bags. Sampling was conducted in five Azorean islands, Madeira, Porto Santo, Deserta Grande and four Cape Verde islands, and complemented by existing specimens on the AZU (Herbário do Departamento de Ciências Agrárias da Universidade dos Açores) and ORT (Instituto Canario de Investigaciones Agrarias) herbaria (see Tables 1 and 2 for sampling information). Selection of target species followed the most recent available checklists for each archipelago (Ginovés et al., 2010; Menezes de Sequeira et al., 2012; Sánchez‐Pinto et al., 2005; Silva et al., 2010). The suspicious individuals of *Musschia angustifolia*, as well as samples from the new *Campanula* species described for Santo Antão (i.e., *C. feijoana* and *C. hortelensis*) were also included and listed on Table 1. Some Macaronesian Campanulaceae sequence data available on GenBank (http://www.ncbi.nlm.nih.gov/genbank) were also used on this study (see Table S1).

**Table 1:** Target native and naturalized *Campanulaceae* species in Macaronesia and their distribution and conservation status on the archipelagos. Distributions are indicated with islands from which samples are represented in this study shown with filled circles and nonsampled islands shown with open circles

*Taxa*	IUCN^a^	Status^b^	Archipelago's species sampling^c^								
			Azores								
			MA	SM	TE	GR	PI	FA	SJ	FL	CO
*Azorina vidalii* (H. C. Watson) Feer	EN	END	●	●	●	○	●	●	○	○	○
*Campanula erinus* L.	NE	NZ	●	●	●	○	○	○	○	○	○
*Lobelia urens* L.	NE	NZ?			●			○		○	
*Trachelium caeruleum* L.	NE	NZ?		●	○			○		○	

*Taxa*	IUCN	Status	Madeira			
			MD	PS	DE	SL
*Campanula erinus* L.	NE	NT	●	●	○	
*Lobelia urens* L.	NE	NT	●			
*Musschia aurea* (L. f.) Dumort.^d^	LC	END	●		●	
*Musschia isambertoi* M. Seq. et al.	NE	END			●	
*Musschia wollastonii* Lowe	EN	END	●			
*Trachelium caeruleum* L.	NE	I	●			
*Wahlenbergia lobelioides* (L. f.) Link subsp. *lobelioides*	NE	NT	●	●	○	○

*Taxa*	IUCN	Status	Canary Islands						
			L	F	C	T	G	P	H
*Campanula erinus* L.	NE	NT?	○	○	○	●	○	●	●
*Wahlenbergia lobelioides* (L. f.) Link subsp. *lobelioides*	NE	NT	●	○	○	○	○	○	○

*Taxa*	IUCN	Status	Cape Verde									
			A	V	N	L	S	B	M	ST	F	BR
*Campanula jacobaea* C. Sm. ex Webb^e^	NE	END	●	●	●					●	○	○
*Campanula bravensis* (Bolle) A. Chev.	NE	END								○	●	●
*Wahlenbergia lobelioides* (L. f.) Link subsp. *lobelioides*	NE	NT?	●	○	●					○	●	

a IUCN status: EN, endangered; LC, least concern; NE, not evaluated.

b Colonization status: END, endemic; NT, native; NZ, naturalized; I, introduced; ?, doubtful status.

c Islands: MA, Santa Maria; SM, São Miguel; TE, Terceira; GR, Graciosa; PI, Pico; FA, Faial; SJ, São Jorge; FL, Flores; CO, Corvo; MD, Madeira; PS, Porto Santo; DE, Desertas; SL, Savage Islands; L, Lanzarote; F, Fuerteventura; T, Tenerife; G, La Gomera; P, La Palma; H, El Hierro; A, Santo Antão; V, São Vicente; L, Santa Luzia; N, São Nicolau; S, Sal; B, Boa Vista; M, Maio; ST, Santiago; F, Fogo; BR, Brava.

d Suspicious individuals of the nonrecognized *M. angustifolia* were collected and included in this study.

e Individuals of *C. jacobaea* collected from Santo Antão correspond to *C. feijoana* and *C. hortelensis*.

**Table 2:** DNA collection codes and localities for the populations used on this study

*Taxa*	Herbarium^a^	DNA codes	Locality	Collector
*Azorina vidalii*	AZB	AV‐SMSV‐04	Azores, São Miguel, São Vicente	Tiago Menezes
	AZB	AV‐SMMO‐06	Azores, São Miguel, Mosteiros	Tiago Menezes
	AZB	AV‐MAPC‐01	Azores, Santa Maria, Ponta do Castelo	Tiago Menezes
	AZB	AV‐TEPM‐01	Azores, Terceira, Porto Martins	Tiago Menezes
	AZB	AV‐TEPJ‐04	Azores, Terceira, Porto Judeu	Tiago Menezes
	AZB	AV‐FAJB‐02	Azores, Faial, Jardim Botânico	Tiago Menezes
	AZB	AV‐PIAM‐02	Azores, Pico, Santo Amaro	Tiago Menezes
	AZB	AV‐PIBR‐05	Azores, Pico, Baixa da Ribeirinha	Tiago Menezes
*Campanula bravensis*	LISC	760	Cape Verde, Fogo, Ribeira do Coxo	M. Romeiras et al.
	LISC	762	Cape Verde, Fogo, Bordeira, Gruta Cruz	M. Romeiras et al.
	LISC	537	Cape Verde, Brava, Cruz da Fajã	John Tennent & Peter Russell
	LISC	5816	Cape Verde, Brava, Cruz da Fajã	John Tennent & Peter Russell
*Campanula erinus*	AZU	5084	Azores, Santa Maria, Praia Formosa	Luís Nunes
	AZB	CE‐SMIP‐01	Azores, São Miguel, Paim	Tiago Menezes
	AZB	CE‐SMCO‐01	Azores, São Miguel, Conceição	Tiago Menezes
	AZB	CE‐MDPN‐01	Madeira, Madeira Island, Porto Novo	M. Sequeira
	AZB	CE‐PSRS‐01	Madeira, Porto Santo, Rocha de Nª. Senhora	M. Sequeira & R. Jardim
	AZB	CE‐PSRS‐02	Madeira, Porto Santo, Rocha de Nª. Senhora	M. Sequeira & R. Jardim
	AZB	CE‐MDCF‐01	Madeira, Madeira Island, Curral das Freiras	M. Sequeira
*Campanula erinus* (*Cont*.)	ORT	13035	Canary Islands, Tenerife, Masca	—
	ORT	13039	Canary Islands, Tenerife, Los Silos	—
	ORT	3462	Canary Islands, La Palma, Sta. Cecilia	—
	ORT	30583	Canary Islands, La Palma, Tijarate	—
	ORT	17168	Canary Islands, El Hierro, El Golfo	—
*Campanula jacobaea*	LISC	4078	Cape Verde, São Nicolau, Pico da Cruz	C. Duarte et al.
	LISC	4128	Cape Verde, São Nicolau, Monte Gordo	C. Duarte et al.
	LISC	1001	Cape Verde, Santiago, Serra da Malagueta	M. Romeiras & M. Carine
	LISC	1000	Cape Verde, Santiago, Serra da Malagueta	M. Romeiras & M. Carine
	LISC	3219	Cape Verde, São Vicente, Monte Verde	M. Romeiras & M. Carine
	LISC	1076	Cape Verde, Santo Antão, Cova	M. Romeiras & M. Carine
	LISC	1095	Cape Verde, Santo Antão, Maroços	M. Romeiras & M. Carine
*Lobelia urens*	AZB	LU‐MDGL‐02	Madeira, Madeira Island, Ginjas	M. Sequeira, A. Sequeira & P. Sequeira
	AZB	LU‐MDFA‐01	Madeira, Madeira Island, Fanal	M. Sequeira, A. Sequeira & P. Sequeira
*Musschia aurea*	AZB	MU‐MDGA‐07	Madeira, Madeira Island, Garajau	M. Sequeira & C. Marques
	AZB	MU‐MDGA‐18	Madeira, Madeira Island, Garajau	M. Sequeira & C. Marques
	AZB	MU‐DEDG‐01	Madeira, Desertas, Deserta Grande	M. Sequeira & C. Marques
	AZB	MU‐DEDG‐02	Madeira, Desertas, Deserta Grande	M. Sequeira & C. Marques
	AZB	MF‐MDPD‐01	Madeira, Madeira Island, Ponta Delgada	M. Sequeira & C. Marques
	AZB	MF‐MDCM‐03	Madeira, Madeira Island, Porto da Cruz	M. Sequeira & C. Marques
*Musschia isambertoi*	AZB	MI‐DEDG‐01	Madeira, Desertas, Deserta Grande	M. Sequeira
*Musschia wollastonii*	AZB	MW‐MDET‐05	Madeira, Madeira Island, Levada do Folhadal	M. Sequeira & C. Marques
	AZB	MW‐MDET‐21	Madeira, Madeira Island, Levada do Folhadal	M. Sequeira & C. Marques
*Trachelium caeruleum*	AZB	TC‐MDFU‐01	Madeira, Madeira Island, Funchal, Alegria	M. Sequeira
	AZB	TC‐SMAP‐01	Azores, São Miguel, Água de Pau	Tiago Menezes
	AZB	TC‐SMFO‐01	Azores, São Miguel, Porto Formoso	Tiago Menezes
*Wahlenbergia lobelioides* subsp. *lobelioides*	AZB	WL‐PSCI‐01	Madeira, Porto Santo, Ilhéu de Cima	M. Sequeira & R. Jardim
	AZB	WL‐MDRB‐02	Madeira, Madeira Island, Ribeira Brava	M. Sequeira
	AZB	WL‐PSPF‐03	Madeira, Porto Santo, Pico Facho	M. Sequeira & R. Jardim
	AZB	WL‐MDPL‐01	Madeira, Madeira Island, Paúl do Mar	M. Sequeira & C. Marques
	ORT	41666	Canary Islands, Lanzarote, Haria Guimate	—
	LISC	2906	Cape Verde, São Nicolau, Monte Gordo	M. Romeiras et al.
	LISC	3597	Cape Verde, Fogo, Chã de Ribeira	M. Romeiras et al.
	LISC	7068	Cape Verde, Santo Antão, Ribeira do Paúl	M. Romeiras et al.

a Herbarium codes: AZB, Herbarium Ruy Telles Palhinha; AZU, Herbarium of Departamento de Ciências Agrárias da Universidade dos Açores; ORT, Instituto Canario de Investigaciones Agrarias (ICIA); LISC, IICT—Herbarium of the Tropical Research Institute. The samples collected for this study were housed in AZB and LISC.

### 2.3 DNA extraction and amplification

DNA was extracted from silica dried leaves using a modified CTAB protocol (3 × CTAB) from Doyle and Dickson (1987). A DNeasy Plant Mini Kit (Qiagen, Crawley, UK) was used to extract DNA from herbarium specimens. DNA samples were kept in sterile deionized water at −20°C, after checking the quality and quantity using the spectrophotometer NanoDrop 2000 (Thermo Fisher Scientific).

Six chloroplast (cpDNA) regions were amplified: *atpB*,* matK*,* petD*,* rbcL*,* trnL‐F* (Crowl et al., 2014) and *psbA‐trnH* (Kress, Wurdack, Zimmer, Weigt, & Janzen, 2005); and one nuclear region (ITS) with the primers of Douzery et al. (1999). All products with the exception of primers, DNA, and pure water were provided by Biotaq PCR Kit (Bioline). The amplification reaction was performed in a T‐Gradient thermocycler (Whatman Biometra). Amplification followed the protocol proposed by Carine et al. (2004), the complete ITS region was amplified with the following thermocycling program: initial denaturation for 1 min at 94°C, followed by 30 cycles of 1 min at 94°C, 1 min at 54°C, 3 min at 72°C, and a final extension step at 72°C for 8 min. For cpDNA markers, PCR conditions were set to an initial denaturation for 2 min at 94°C, followed by 30 cycles of 30 s at 94°C, 50°C for 30 s, and 1‐min extension at 72°C, with a final extension of 5 min at 72°C.

The DNA fragments resulting from amplification were separated on agarose gel, 0.7%–1% in TBE buffer, stained with SafeView Classic Nucleic Acid Stain and were visualized with Visidoc‐IT imaging system (UVP). A molecular marker 50–2,000 base pairs (Sigma‐Aldrich) was used as reference. The amplification products were sequenced by STABVIDA, Lda (Portugal).

### 2.4 Phylogenetic analysis

Sequence data were assembled, edited, and aligned using Geneious ver. 7.0.6 (Biomatters Ltd.) and the Geneious alignment algorithm. The alignments were then inspected and manually optimized.

Analyses were conducted separately for each marker (ITS, *atpB*,* matK*,* psbA‐trnH*,* petD*,* rbcL*, and *trnL‐F*) and combined (all six chloroplast regions or all seven nuclear and plastidial loci). Several *Cyphia* P.J. Bergius species were used as out‐group, such as *C. elata* Harv., *C. comptonii* Bond, *C. decora* Thulin, *C. rogersii* S. Moore, and *C. tysonii* E. Phillips (Crowl et al., 2014). The obtained tree topology for each marker was then compared in order to detect discrepancies.

Maximum‐parsimony (MP) analysis were conducted in PAUP* ver. 4.0 beta 10 (Swofford, 2003). The analysis used 1,000 heuristic searches, random stepwise addition, and TBR branch swapping. A strict consensus tree was calculated.

jModelTest ver. 2.1.3 (Darriba, Taboada, Doallo, & Posada, 2012) was used to determine the best fitting model of sequence evolution based on the Akaike information criterion. In general, GTR (or its variations) was either the best model estimated or was among the top three selected models. A maximum‐likelihood (ML) analysis was conducted using RAxML‐HPC2 ver. 7.4.4 (Stamatakis, Hoover, & Rougemont, 2008) and the default settings on the CIPRES Science Gateway (Miller, Pfeiffer, & Schwartz, 2010) with 1,000 bootstraps and a partitioned dataset for the combined matrix.

Bayesian phylogenetic inference (BI) was obtained using MrBayes v. 3.2 (Ronquist et al., 2012) on CIPRES Science Gateway (Miller et al., 2010) using the GTR model. The analyses were performed with two simultaneous runs of Metropolis‐coupled Markov chains Monte Carlo (MCMCMC), each with four parallel Markov chains. Each chain was performed for 10 million generations and, starting with a random tree, one tree was saved every 100th generation. For other parameters, the default settings of the program were left unchanged. The program Tracer v1.6 (Rambaut, Suchard, Xie, & Drummond, 2014) was used to assess the stationarity of and convergence between the runs and determine the burn‐in. After discarding the burn‐in trees, a 70% majority rule consensus tree was calculated and posterior probabilities (PP) estimated. In order to assess tree topologies and to visualize the trees with node supports, TreeGraph 2 (Stöver & Müller, 2010) was used.

The NeighborNet algorithm (Bryant & Mouton, 2004) as implemented in SplitsTree v4.0 (Huson & Bryant, 2006) was used with the default settings to visualize possible incongruences in the dataset. This method reduces the assumption that evolution follows a strictly bifurcating path and allows for the identification of reticulated evolution or incomplete lineage sorting among the dataset.

Levels of variation of target species were determined for each Macaronesian region, and statistical parsimony networks (Templeton, Crandall, & Sing, 1992) were produced with PopART ver. 1.7 using the TCS Network algorithm (Clement, Snell, Walke, Posada, & Crandall, 2002). The chloroplast markers included in the alignments were selected to obtain the highest possible number of haplotypes (Tables S1 and S2).

The new 310 sequences were submitted to GenBank (accessions numbers are listed in the appendix, Table S2).

### 2.5 Divergence time estimation

Divergence times within the Campanulaceae family were estimated using the Bayesian MCMC algorithm implemented in BEAST v2.4.6 (Drummond, Suchard, Xie, & Rambaut, 2012). For this analysis, we used the combination of the ITS and three cpDNA markers (*matK*,* rbcL*, and *petD*), and The GTR model of sequence substitution was used for the dataset. To calibrate our phylogenetic tree, we used fossil seeds identified as *Campanula sp*. and *Campanula paleopyramidalis* dating from the Miocene (ca. 17–16 Mya; Lancucka‐Srodoniowa, 1977, 1979) and applied to the node representing the last common ancestor of *C. pyramidalis* and *C. carpatica*. Therefore, a prior was applied to the root of the phylogenetic tree of this study and We used date ranges from the 95% highest posterior densities from Bell et al. (2010) to constrain the root of the tree (65–56 Mya). A relaxed lognormal molecular clock was used for all partitions, the Yule process was implemented for the two prior with a uniform model, and a random tree was used as the starting tree. The Bayesian MCMC was run for 10^8^generations, with one tree sampled every 1,000 generations.

To obtain more accurate divergence times for Macaronesian lineages, we used the coalescent species tree method StarBEAST2 in BEAST v2.4.6 (Drummond et al., 2012). These analyses were conducted for (1) *Azorina*,* C. jacobaea* and *C. bravensis*, (2) *Musschia*, and (3) *W. lobelioides* subsp. *lobelioides*. We used two combination of the ITS and four cpDNA markers (*matK*,* rbcL*,* trnL‐F* and *petD*) and The GTR model of sequence substitution was used for all partitions. To calibrate the phylogenetic trees, we used the following secondary calibrations obtained in the first BEAST analysis, described on Table 3: for *Azorina* and Cape Verde *Campanula* were used the nodes C1 and C2; for *Musschia* were used the nodes C7, C8, and C9; and for *W. lobelioides* subsp. *lobelioides* was used the node C11. An uncorrelated exponential clock and the Yule model were implemented for the prior with a uniform model applied. All other parameters were left as default in StarBEAST2. The MCMC was run for 10^8^ generations, with one tree sampled every 5,000 generations.

**Table 3:** Estimation of divergence dates of Macaronesian accessions within Campanulaceae using BEAST as means and 95% highest posterior densities (HPD), in millions of years (Mya)

	Figure 8	Mean age (Mya)	Upper 95% HPD value	Lower 95% HPD value
C1	*Azorina* and Cape Verde *Campanula* group	17.91	25.37	11.38
C2	Cape Verde *Campanula*	13.24	19.34	7.81
C3	*Campanula bravensis* and *C. jacobaea*	1.06	2.39	0.12
C4	*Azorina* group and *Campanula erinus* clade	25.42	34.42	16.3
C5	European and Macaronesian *Campanula erinus*	3.42	6.41	1.03
C6	*Campanula* group and *Trachelium*	45.43	58.46	32.45
C7	*Musschia* clade and *Campanula peregrine*/*C. lactiflora* clade	16.02	25.21	7.8
C8	*Campanula peregrina* and *C. lactiflora*	11.64	19.51	4.73
C9	*Musschia* clade	5.0	8.77	1.94
C10	*Wahlenbergia lobelioides* subsp. *lobelioides* and *W. hederacea*	31.38	46.49	16.55
C11	*Wahlenbergia lobelioides* subsp. *lobelioides* clade	2.27	4.16	0.79
C12	*Canarina canariensis* and *Platycodon grandiflorus*	16.43	25.92	8.01

These analyses were conducted three independent times. Tracer v1.6 (Rambaut et al., 2014) was used to assess convergence and correct mixing of all parameters by visually inspecting the log traces and estimating the effective sample size (ESS) of each parameter. The ESS values were high for all the analyses, except for *Wahlenbergia* although the topology is in agreement with the phylogenetic analysis and BEAST estimates. Results from the three runs were combined with LogCombiner v2.4.6 (Drummond et al., 2012), after discarding the 10 first % of each analysis as burn‐in. The remaining trees were summarized using a maximum‐clade‐credibility target tree in TreeAnnotator v2.4.6 (Drummond et al., 2012), as well as Bayesian PP, MEDIAN/MEAN height, and the 95% highest posterior density heights interval (95% HPD) of each node. All computational analyses were performed in the CIPRES Gateway cloud servers (Miller et al., 2010).

## 3 RESULTS

### 3.1 Phylogenetics

Parsimony analysis of the concatenated data matrix generated was rooted with *Cyphia* spp. for the seven markers used in this study: the ITS and the chloroplast markers *matK*,* rbcL*,* psbA‐trnH*,* trnL‐F*,* petD*, and *atpB* (parameters of the best parsimonious trees are described on Table S3).

Topological discrepancies between the nuclear and cp data were obtained resulting in a concatenated ITS+cp tree less resolved (see Supporting Information for trees obtained for each cp marker and for the ITS+cp matrix).

**Figure 2:**
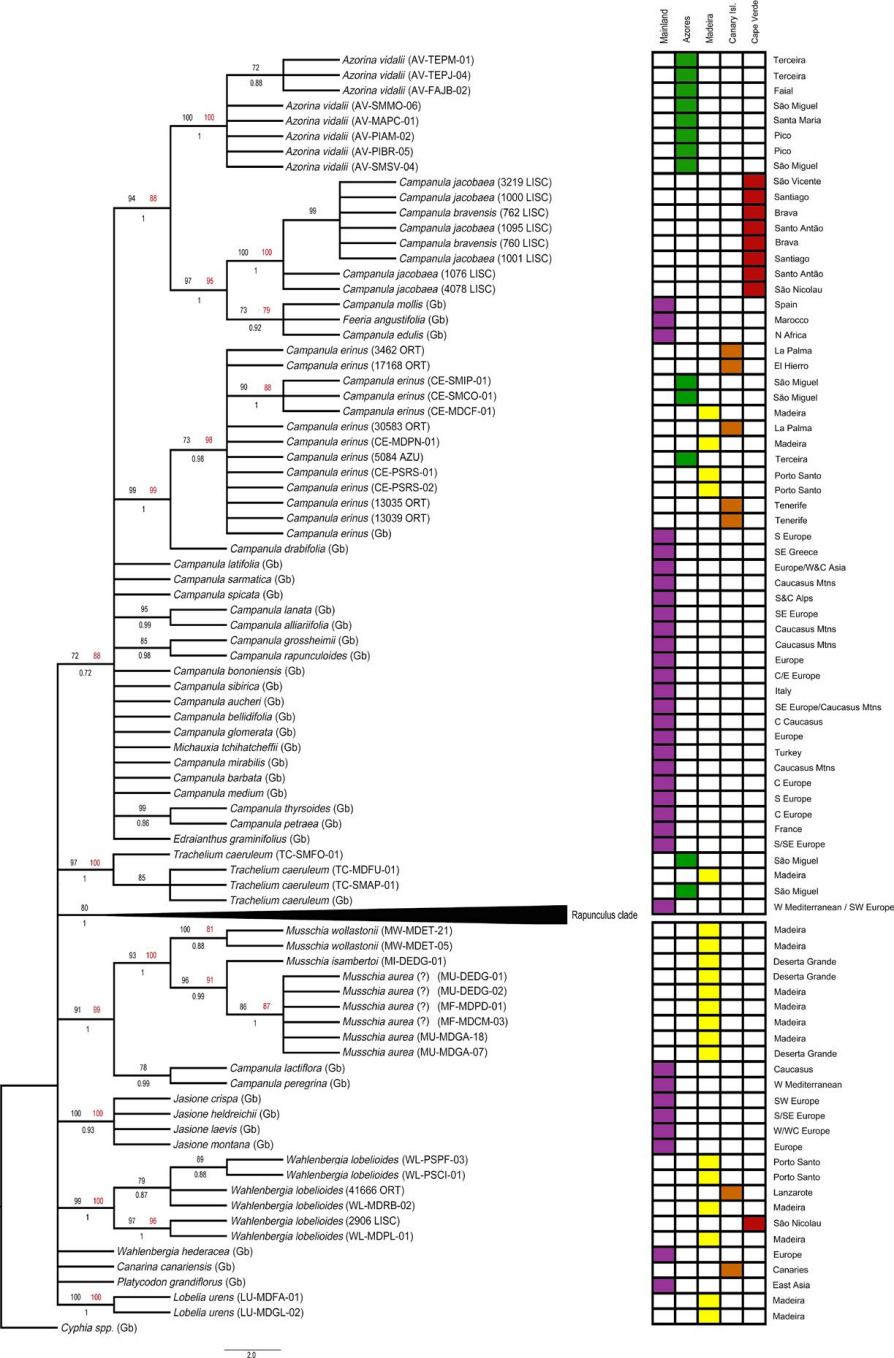
ITS phylogeny. Best tree from maximum‐likelihood analysis. Numbers above branches (≥70%) are maximum‐likelihood (black) and maximum‐parsimony bootstrap values (red). Number bellow branches (≥0.70) are Bayesian posterior probabilities. Sequences of *taxa* labeled with “(Gb)” were obtained on GenBank (Table S2)

**Figure 3:**
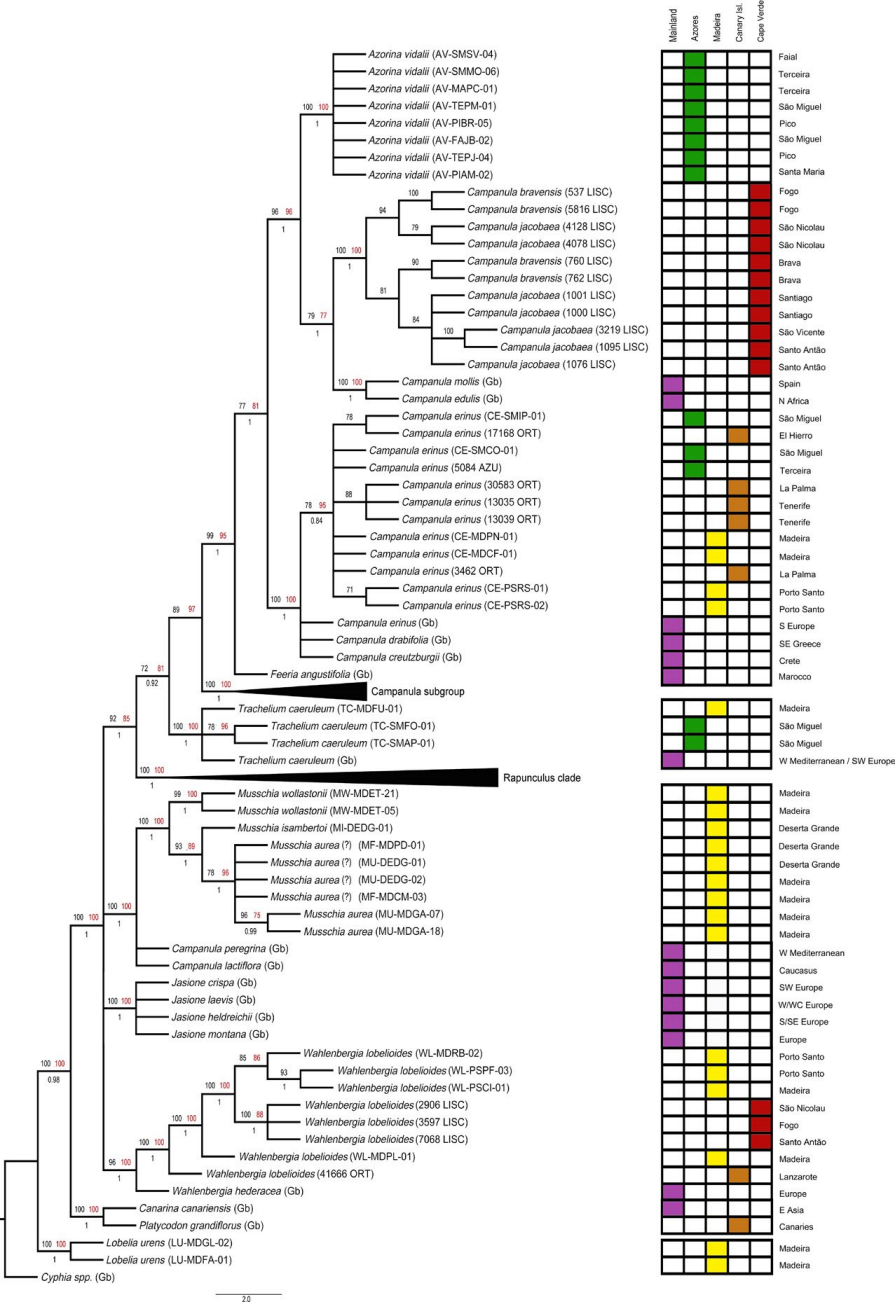
Plastid phylogeny. Best tree from maximum‐likelihood analysis of combined plastid dataset: *matK*,* rbcL*,* psbA‐trnH*,* trnL‐F*,* petD*, and *atpB*. Numbers above branches (≥70%) are maximum‐likelihood (black) and maximum‐parsimony bootstrap values (red). Number bellow branches (≥0.70) are Bayesian posterior probabilities. Sequences of *taxa* labeled with “(Gb)” were obtained on GenBank (Table S2)

In our phylogenetic analyses, *Azorina vidalii* is a well‐supported monophyletic group (ML = 100%; MP = 100%; PP = 1; Figures 2 and 3) and the accessions were polymorphic in two ITS nucleotide positions. It is possible to distinguish a subgroup in the ITS tree with medium bootstrap support (ML = 72%, PP = 0.88; Figure 2) composed by three accessions from Terceira and Faial islands.

*Campanula* species endemic in Cape Verde form a well‐supported clade. Although the markers used in this study did not provide clear distinction among described taxa, several geographically distinct clades were obtained with the cp dataset (Figure 3) for Fogo, São Nicolau, Brava, and also for Santiago with the ITS+cp dataset (Fig. S1). Furthermore, the clade composed by the Cape Verde *Campanula* endemic species plus *Campanula mollis*,* C. edulis*, and *Feeria angustifolia* is sister to the *A. vidalii* clade and is well supported on the ITS tree and on the combined cp tree but without *F. angustifolia* (Figures 2 and 3).

*Campanula erinus* resulted in a monophyletic clade. The Macaronesian accessions were distinct from South Europe, forming a medium‐supported clade on the combined cp tree (ML = 78%, MP = 95%, PP = 0.84; Figure 3) and on the ITS+cp tree (Fig. S1). A well‐supported clade was obtained on the ITS tree (ML = 90%, MP = 88%, PP = 1; Figure 2), separating S. Miguel and Madeira from the remaining Macaronesian accessions.

*Trachelium caeruleum*, considered to be an introduced species in Madeira and Azores, grouped as monophyletic with strong support on all analyses, showing a distinctiveness of the Azorean accessions on the combined cp tree, albeit with medium support (ML = 78%, MP = 96%; Figure 3). Additionally, the Madeiran accession is showed as distinct from the others (Fig. S2) and in the concatenated ITS+cp tree although with low support values (below 70%) on all analyses.

The *Musschia* clade, composed by *M. wollastonii*,* M. isambertoi*, and *M. aurea*, is well supported (ML = 93/100%, MP = 100%, PP = 1; Figures 2 and 3), and each species is monophyletic. *Musschia aurea* is sister to *M. isambertoi*, and this group is sister to *M. wollastonii* (Figures 2, 3 and S1). Furthermore, Garajau population of Madeira island is distinguishable from all other samples of *M. aurea* on the combined cp (ML = 96%, MP = 75%, PP = 0.99; Figure 3) tree.

*Wahlenbergia lobelioides* subsp. *lobelioides* forms a monophyletic group, and two generally well‐supported sister clades were retrieved by the combined cp analyses (Figure 3): (1) one accession from Madeira with all the accessions from Porto Santo (ML = 85%, MP = 86%, PP = 1; Figure 3); and (2) all Cape Verde accessions (ML = 100%; MP = 88%; PP = 1; Figure 3). Within the Madeira group, the populations of Porto Santo group distinctively with high support (ML = 93%, PP = 1; Figure 3). *Lobelia urens* is a fully supported monophyletic group, sister to the other clades in all the analyses.

**Figure 4:**
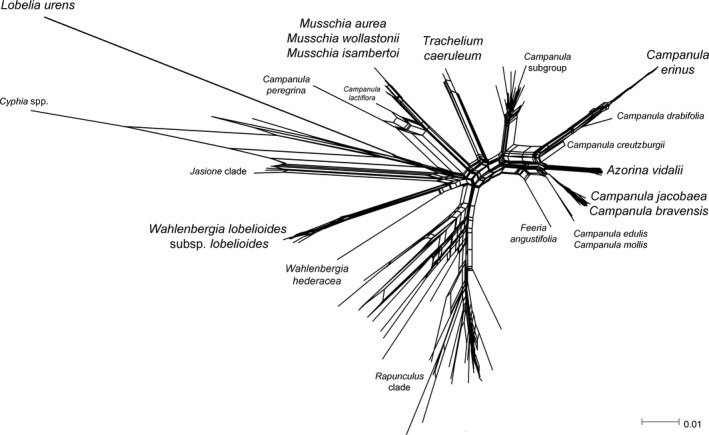
NeighborNet phylogenetic network of the concatenated ITS + cp dataset of Campanulaceae

The NeighborNet algorithm applied to the concatenated ITS+cp dataset revealed a high degree of conflicting phylogenetic signals at the divergences of *Wahlenbergia*,* Musschia*,* Trachelium*, and *Campanula*, evidenced by the substantial number of loops found in these points of the phylogenetic network (Figure 4).

**Figure 5:**
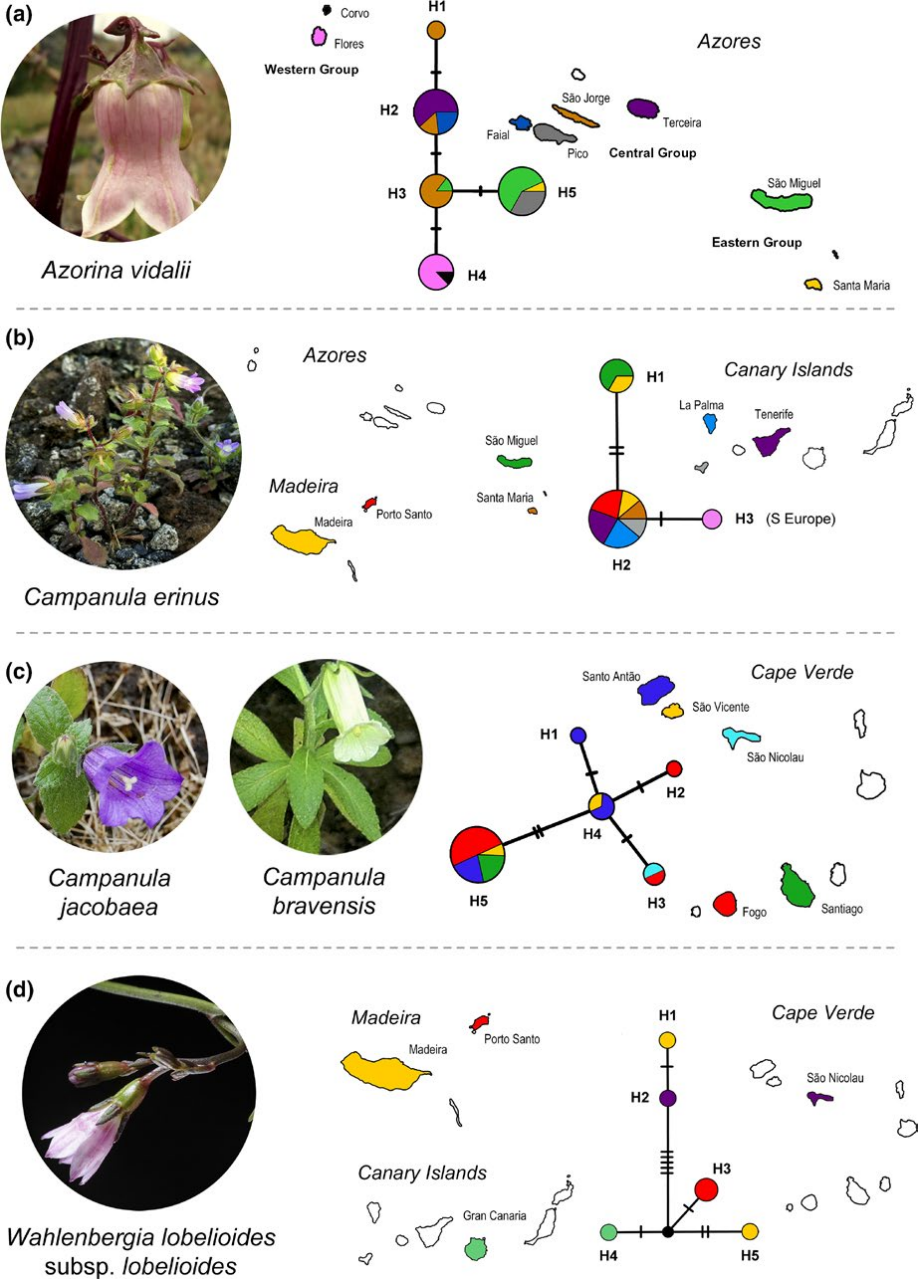
ITS networks for *Azorina vidalii* (a), *Campanula erinus* (b), *C. bravensis* (c—H2, H3, H5), *C. jacobaea* (c—H1, H3, H4, H5), and *Wahlenbergia lobelioides* subsp. *lobelioides* (d). For each taxon, colors of islands match those used in the corresponding network. White indicates not sampled. Size of circles is proportional to the number of individuals with each ribotype

Regarding haplotype diversity, for *A. vidalii* (Figure 5a), one ribotype was found to be endemic to São Jorge (H1). The other four ribotypes were shared between islands: H2 being endemic to the central group, H3 is shared among São Jorge and São Miguel, H4 is endemic to the western group, and H5 is shared by plants sampled from eastern group and some plants sampled from Pico.

In the *C. erinus* ribotype network (Figure 5b), H1 is shared between São Miguel and Madeira island, H2 is shared among Canary Islands, Madeira, and Santa Maria island from the Azores, and H3 is related to the sample of the mainland, from southern Europe.

Regarding the ITS network obtained for the Cape Verde *Campanula* (Figure 5c), H1 is a single‐island endemic for the new Campanula species from Santo Antão, as well H2 for Fogo of *C. bravensis*; H4 is shared between Santo Antão and for *C. jacobaea* from São Vicente; H3 and H5 are composed by both species and shared between islands.

**Figure 6:**
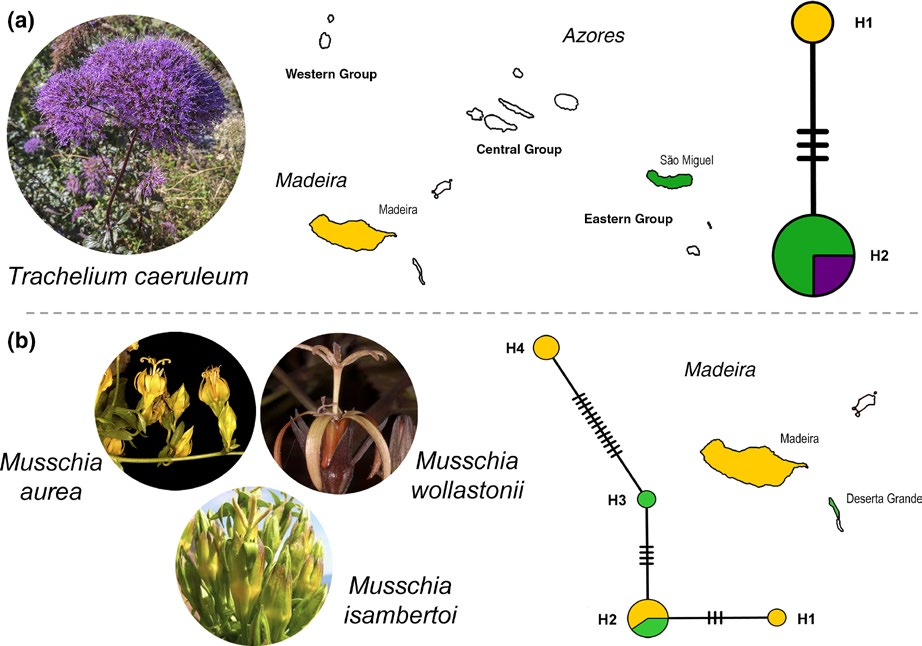
ITS and cp networks for *Trachelium caeruleum* (a), *Musschia aurea* (b—H1, H2), *M. isambertoi* (b—H3), and *M. wollastonii* (b—H4). For each taxon, colors of islands match those used in the corresponding network. White indicates not sampled. Purple in AH2 is related to a Western Mediterranean/South‐western Europe accession. Size of circles is proportional to the number of individuals with each ribotype

For *T. caeruleum* the network (Figure 6a) included H1, a single‐island haplotype from Madeira island and H2, shared between São Miguel (Azores) and the Western Mediterranean/South‐western Europe accession.

For *Musschia*, the haplotype network obtained (Figure 6b) was composed by two *M. aurea* haplotypes (H1 and H2), with H1 being specific to Madeira Island, and by one *M. isambertoi* haplotype (H3) in Deserta Grande, while H4 is a *M. wollastonii* haplotype specific to Madeira Island.

**Figure 7:**
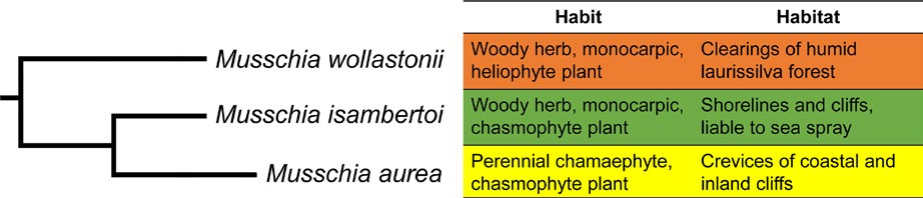
Phylogeny of *Musschia* genus with habit and habitat of *Musschia wollastonii*,* M. isambertoi*, and *M. aurea*

Regarding the habit and habitat of *Musschia* (Figure 7), *M. wollastonii* and *M. isambertoi* are woody monocarpic herbs, which are present in clearings of laurissilva forest and in the shorelines and cliffs, respectively. *Musschia aurea* is a woody perennial chamaephyte found as a chasmophyte in crevices of coastal and inland cliffs.

The haplotype network for *W. lobelioides* subsp*. lobelioides* (Figure 5d) retrieved five single‐island endemic haplotypes: H1 and H5 from Madeira Island, H3 from Porto Santo, H4 from the Canary Islands, and H2 from Cape Verde.

### 3.2 Divergence time estimation

**Figure 8:**
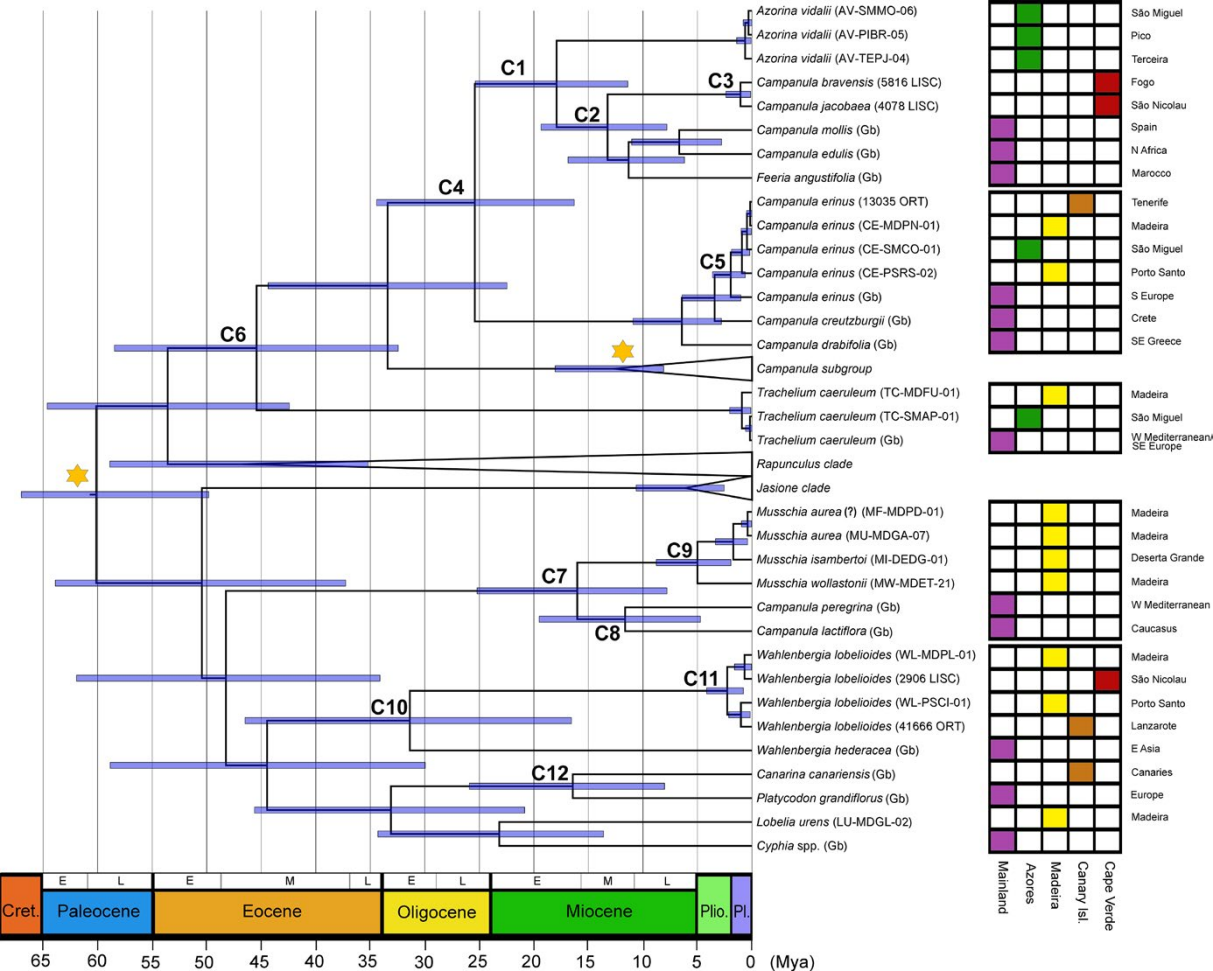
Maximum‐clade‐credibility (MCC) time‐calibrated tree of Campanulaceae species for Macaronesia inferred and dated with a Bayesian analysis implemented in Beast, based on the concatenated dataset of ITS and cpDNA markers (*matK*,* rbcL* and *petD*), illustrating the estimated divergence ages at selected calibrated nodes. The geographic origin of each specimen is provided (right side) with a color code for continental areas and for the Macaronesian archipelagos. C1–C9 as described in Table 3. Mya, million years ago

**Figure 9:**
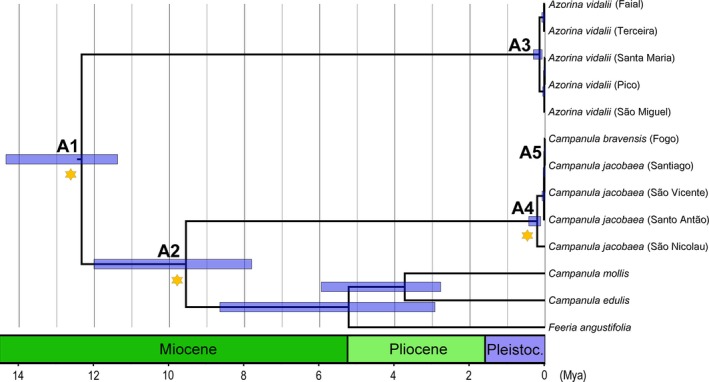
Maximum‐clade‐credibility (MCC) time‐calibrated tree of *Azorina* and Cape Verde *Campanula* in Macaronesia inferred and dated using a multispecies coalescent method (StarBEAST2), based on the two partitions dataset: ITS and cpDNA markers (*matK*,* rbcL*,* trnL‐F* and *petD*), illustrating the estimated divergence ages at selected calibrated nodes. A1–A5 as described in Table 4. Mya, million years ago

Date estimates for nodes within the Campanulaceae family in Macaronesia are presented in Figure 8 (see C1 to C9, Table 3). Our analysis indicates that the *Azorina* must have diverged from *Campanula* group composed by the Cape Verde endemics and their sister species *C. mollis*,* C. edulis* and *F. angustifolia* (A1; Figure 9, Table 4) around 12.34 Mya. Within this group, the divergence between the Cape Verde *Campanula* and their sister species (C2, Figure 8, Table 3) was estimated to have occurred at 9.56 Mya (A2; Figure 9, Table 4). The split between *C. bravensis* and *C. jacobaea* (C3; Figure 8, Table 3) may have occurred recently, in the Pleistocene, at 1.06 Mya (95% HPD: 2.39–0.12 Mya). With a StarBEAST2 analysis, it was possible to understand that cladogenesis in *C. jacobaea* happened at 0.21 Mya (A4, Figure 9, Table 4) and *C. bravensis* diverged from it at roughly 0.01 Mya (A5, Figure 9, Table 4).

**Table 4:** Estimation of divergence dates of Macaronesian accessions within Campanulaceae using StarBEAST2 as means and 95% highest posterior densities (HPD), in millions of years (Mya)

	Figures 9, 10, 11	Mean age (Mya)	Upper 95% HPD value	Lower 95% HPD value
*Azorina* and Cape Verde *Campanula* clade				
A1	*Azorina* and *Campanula*	12.34	14.35	11.38
A2	Cape Verde *Campanula* and sister species	9.56	12.01	7.81
A3	*Azorina* clade	0.15	0.31	0.08
A4	*Campanula jacobaea* clade	0.21	0.43	0.12
A5	*C. jacobaea* and *C. bravensis* (Fogo island)	0.01	0.03	0.0
*Musschia* clade				
M1	*Campanula peregrina/C. lactiflora* from *Musschia* clade	8.27	9.28	7.8
M2	*Musschia wollastonii*	3.40	4.82	1.94
M3	*Musschia isambertoi* and *M. aurea*	0.07	0.18	0.006
M4	*Musschia aurea* and *M. aurea* (?)	0.01	0.02	0.0
*Wahlenbergia lobelioides* clade				
W1	Canaries and Madeira/Porto Santo/Cape Verde clade	0.87	1.06	0.79
W2	Madeira archipelago and Cape Verde/Madeira	0.07	0.18	0.01
W3	Madeira island and Porto Santo	0.04	0.11	0.002
W4	Cape Verde and Madeira island	0.01	0.03	0.0001

The clade containing *C. erinus* (C4, Figure 8, Table 3) have diverged from the remaining *Campanula* species about 25.42 Mya, and the Macaronesian clade (C5, Figure 8, Table 3) diverged from the south European *C. erinus* roughly at 3.42 Mya (95% HPD: 6.41–1.03 Mya). The divergence between this group and *T. caeruleum* (C6, Figure 8, Table 3) occurred in the Eocene, at 45.43 Mya.

**Figure 10:**
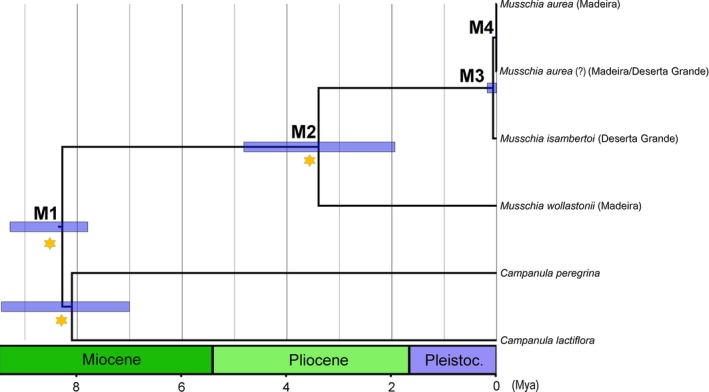
Maximum‐clade‐credibility (MCC) time‐calibrated tree of *Musschia* species inferred and dated using a multispecies coalescent method (StarBEAST2), based on the two partitions dataset: ITS and cpDNA markers (*matK*,* rbcL*,* trnL‐F*, and *petD*), illustrating the estimated divergence ages at selected calibrated nodes. M1–M4 as described in Table 4. Mya, million years ago

More recently, in the Miocene, a split between *C. peregrina* and *C. lactiflora* from *Musschia* (M1, Figure 10, Table 4) happened around 8.27 Mya (95% HPD: 9.28–7.8 Mya).

Cladogenesis in *Musschia* started in the middle of Pliocene at roughly 3.4 Mya (M2, Figure 10, Table 4) giving origin to *M. wollastonii*. Recently, in the Pleistocene, divergence between *M. isambertoi* and *M. aurea* occurred at ca. 0.07 Mya (M3, Figure 10, Table 4). Also, our results estimate a divergence between *M. aurea* and the suspicious individuals from Madeira island (the putative *M. angustifolia*) and Deserta Grande at 0.01 Mya (M4, Figure 10, Table 4).

**Figure 11:**
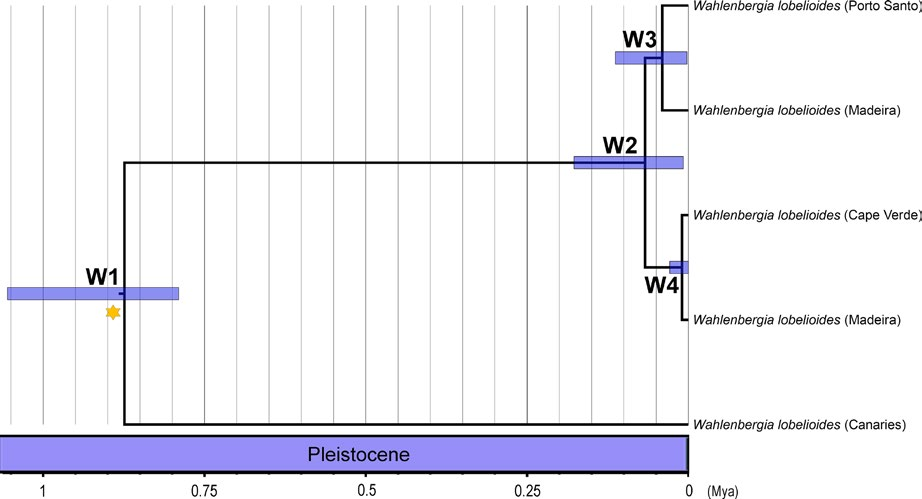
Maximum‐clade‐credibility (MCC) time‐calibrated tree of *Wahlenbergia lobelioides* subsp. *lobelioides* for Macaronesia inferred and dated using a multispecies coalescent method (StarBEAST2), based on the two partitions dataset: ITS and cpDNA markers (*matK*,* rbcL*,* trnL‐F*, and *petD*), illustrating the estimated divergence ages at selected calibrated nodes. W1–W4 as described in Table 4. Mya, million years ago

Regarding *Wahlenbergia*, the divergence from *W. hederacea* (C8, Figure 8, Table 3) started in the beginning of the Oligocene, around 31.38 Mya, and the speciation of the Macaronesian endemic *W. lobelioides* subsp. *lobelioides* occurred in the Pleistocene, at ca. 0.87 Mya (W1, Figure 11, Table 4). It is possible to distinguee two groups: (1) the divergence between Madeira and Porto Santo at 0.04 Mya (W3, Figure 11, Table 4); and (2) the divergence between Cape Verde and Madeira at ca. 0.01 Mya (W4, Figure 11, Table 4).

The clade formed by *Platycodon grandiflorus* and *C. canariensis* (C9, Figure 8, Table 3) started their divergence roughly at 16.43 Mya (95% HPD: 25.92–8.01 Mya).

## 4 DISCUSSION

### 4.1 Phylogenetic analyses

Recent studies conducted within the Campanulaceae family have made significant progresses toward a robust phylogenetic hypothesis of the group (Crowl et al., 2014; 2016). These authors concluded that previous studies including ITS have shown significant limitation in resolving species level relationships and providing accurate information on the placement of several genera (e.g., *Jasione* and *Musschia*). In our study, these two genera resolved as monophyletic but a polytomy still persisted, however, our combined chloroplast markers resulted in a well‐resolved tree regarding the relationships between the other target clades. Hybridization is an evolutionary force common in plants (*e.g*. Payseur & Rieseberg, 2016) and within the Campanulaceae family is a likely cause of the discrepancy between the nuclear and chloroplast trees (Wendling et al., 2011). The significant quantity of loops that can be found in the NeighborNet network in this study, further leads to the hypothesis of early hybridization between different species along the evolutionary story of Campanulaceae, and a similar case of was reported by Romeiras, Vieira, et al. (2016) for the Macaronesian Beta *ssp*. (Amarathaceae family). This may be related to the high controversial taxonomy based in several phylogenetic studies with paraphyly and polyphyly observed in some genera that were reported by recent studies (e.g., Cosner et al., 2004; Crowl et al., 2014, 2016; Eddie et al., 2003; Haberle et al., 2009; Roquet et al., 2008).

Our results support a phylogenetically close relation of *Azorina* clade to Cape Verde and Mediterranean/African/West Asian *Campanula*, which is in accordance with Haberle et al. (2009), Olesen et al. (2012), and Crowl et al. (2014). It thus seems that *A. vidalii*,* C. jacobaea* and *C. bravensis*,* C. mollis*,* C. edulis*, and *Feria angustifolia* may share a common ancestor in North Africa.

Variability between the *A. vidalii* accessions collected in the Azores was detected in the ITS region. It was possible to distinguish (ITS region) accessions of three populations of *A. vidalii* from two islands of the central group (Terceira and Faial). This is indicative that there are ongoing evolution processes happening in *A. vidalii* for the central group in the Azores. Furthermore, the network obtained showed a single‐island endemic ribotypes (São Jorge, Western Group and Central Group), and two others shared between islands of the Central and the Eastern groups. A result very close to the one obtained by Schaefer et al. (2011), although the endemic ribotype reported for Pico appears now shared with the Eastern Group.

Watson (1870) described that *A. vidalii* was collected in 1842 for the first time on an islet off the island of Flores. However, upon reaching the island, they found no evidence of the plant. Trelease (1897), in an expedition to the Azores in the 1890s, was surprised to find such abundance of *A. vidalii* in Flores and suggested it probably was endemic to Flores Island and subsequently had been introduced to the other islands. Schaefer et al. (2011) defended that it is unlikely this species would be restricted to Flores prior to human impact with a native distribution certainly extending to the central subarchipelago. Although Schaefer et al. (2011) indicated the existence of a ribotype shared between the Eastern Group and Flores Island, our molecular data could not confirm it. Considering the number of ribotypes obtained in this study for the Central Group, and in the Schaefer et al. (2011), the present distribution of *A. vidalii* may have resulted from dispersal events from the Central group to the Eastern and Western groups.

The long distances between the Macaronesian archipelagos may be responsible for high levels of diversity found on these archipelagos, acting as effective barriers to dispersal and promoting allopatric speciation (Schaefer et al., 2011). However, within‐island diversification (i.e., in *A. vidalii*) might be due to complex topographies and long eruptive episodes that happened on these islands (Borges Silva et al., 2016; Brown, Hoskisson, Welton, & Baez, 2006; Dias, Moura, Schaefer, & Silva, 2016; Juan, Emerson, Oromí, & Hewitt, 2000; Silva et al., 2015).

Considering the Cape Verde diversity, two endemic species (i.e., *Campanula hortelensis* and *C. feijoana*) were recently described to Santo Antão Island; however, a split from *C. jacobaea* was not clear in this study and other molecular studies are needed to clarify the diversification of the *Campanula* species within this archipelago. Nevertheless, there seems to be some variability within *C. jacobaea*, namely from São Nicolau and Santiago Islands, as well as an endemic haplotype for Santo Antão. *Campanula bravensis* shows an endemic haplotype for Fogo Island. The weak groups obtained within Cape Verde taxa may be the result of recurrent gene flow between the two congeneric taxa or recent island colonization leading to incipient differentiation within these species and incomplete lineage sorting. The latter is in agreement with the incongruent phylogenetic signals obtained between nuclear and chloroplast data. Although hybridization episodes may also be involved, most loops observed in the phylogenetic network analysis seem to be ancient possibly related to earlier hybridization events with other closely related taxa such as an ancestral of *Azorina*.

Regardless of the evolutionary forces that may be acting in the Cape Verde *Campanula*, our results indicate that a thorough taxonomic revision should be conducted in order to determine if the geographic grouping obtained is reflected in any way at the morphological level.

For the first time, a phylogenetic study of Campanulaceae included a complete sampling of all *Musschia* species. A clear separation between *M. wollastonii*,* M. isambertoi*, and *M. aurea* was obtained and provided molecular support for the morphological differences already described between the three species (Dumortier, 1823; Lowe, 1856; Sequeira, Jardim, Silva, & Carvalho, 2007). Dumortier (1823) did a distinction between *M. aurea* and a fourth species, *M. angustifolia*, based on their habitat: coastal and inland, respectively. However, the existence of *M. angustifolia* on Madeira has not been widely recognized (Sequeira et al., 2007). During the sampling conducted for this study, it was observed that the morphology was slightly different in some populations of *M. aurea*. These plants, included in the study, show narrow leaves and smaller flowers which correspond to the taxon previously described by Dumortier (1823) as *M. angustifolia*. The molecular data supported a distinction of the *M. aurea* population from Garajau but not for the putative individuals with distinct morphology. The haplotype network showed two ribotypes for *M. aurea* one being specific to the Garajau population. A thorough morphological revision is needed to further ascertain the distinctiveness of *M. angustifolia* obtained in this study at the molecular level.

Regarding the native species, our results seem to indicate the presence of distinct endemic groups within the Macaronesian archipelagos and future taxonomic revisions should be conducted. Particularly, *C. erinus* Macaronesian accessions appear to be distinctive from the South European. This may suggest a separate taxon for Macaronesia with some incipient differentiation between archipelagos and among islands, as is the case of Porto Santo and several islands of the Canaries. Moreover, the chloroplast data and haplotype network of *W. lobelioides* subsp. *lobelioides* seem to indicate the presence of distinct endemic groups in the archipelagos of Madeira, Canary Islands, and Cape Verde. In the past studies (e.g., Crowl et al., 2014; Cupido, 2009; Eddie et al., 2003; Haberle et al., 2009; Olesen et al., 2012 and Roquet et al., 2009), *Wahlenbergia hederacea* was placed related within the *Jasione* clade. Eddie and Cupido (2014) proposed a new generic name for *W. hederacea* as *Hesperocodon hederaceus* (L.) Eddie & Cupido, based on the last molecular studies and its main morphological characteristics, which are fundamentally campanuloid. Eddie and Cupido (2014) concluded that the capsule dehiscence mechanism of *W. hederacea* is essentially wahlenbergioid and differs from most campanuloids, considered together with *Feeria* and *Jasione* as “transitional” genera due to these characteristics intermediate between typical wahlenbergioids and typical campanuloids. However, our study, *W. hederacea* is within *Wahlenbergia* clade, being sister of *W. lobelioides* subsp*. lobelioides*. The Macaronesian endemic species may be the missing link that could provide the right relationship between *W. hederacea* and its respective clade. There can also be seen a quantity of loops on Figure 6 that report an ancestral hybridization between *W. hederacea* and *W. lobelioides* subsp. *lobelioides*.

In what concerns the Madeiran *T. caeruleum* haplotype, the molecular patterns detected might be linked to a man‐mediated founder effect (Frankham, 1997), considering records of the introduction of this species in the Madeira island ca. 1840 (Lowe, 1868). The intensity of the founder effect is related to the number of plants introduced and to the genetic diversity present in the population of origin (Frankham, 1997), and thus, it will be important to expand the sampling of *T. caeruleum* with samples from the Mediterranean area of origin. Regarding the Azorean *T. caeruleum*, an in‐depth morphological revision should be conducted, due to the separate grouping obtained in some analyses for the Azores, and considering that Trelease (1897) refers for the island of Flores that inhabitants said to took inland plants to their gardens, being thus possible to be a native species for the archipelago.

Unfortunately, no accessions of *L. urens* were found on GenBank and the herbarium specimens from the Azores did not result in readable sequences. However, specimens collected on Madeira archipelago gave an indication of the position of this species within the Campanulaceae family, as sister to all other analyzed taxa.

The information provided in this study is an important step toward future taxonomic studies within Macaronesian Campanulaceae and a stepping stone to future revisions and possible circumscription of new native and endemic taxa.

### 4.2 Diversification of Campanulaceae in Macaronesia

The Macaronesian flora shows an important connection with Southwest Europe and the Northwest African flora, being the last one the main source of colonization events in many Macaronesian lineages (Sanmartín, Anderson, Alarcón, Ronquist, & Aldasoro, 2010).

The estimation of divergence times in this study provides information about the diversification of relevant lineages of Campanulaceae species in Macaronesia, such as *Azorina*,* Musschia*,* C. jacobaea*,* C. bravensis*, and *W. lobelioides* subsp. *lobelioides*, revealing that diversification was quite recent, during the late Pliocene and Pleistocene: a recurrent pattern reported for this biogeographic region (e.g., Bateman, Rudall, & Moura, 2013; Romeiras, Vieira, et al., 2016; Romeiras et al., 2011).

According with our results, the common ancestor between *Azorina* and the clade containing the Cape Verde endemics *Campanula* started to diverge in the Medium Miocene (12.34 Mya). García‐Aloy et al. (2017) obtained a divergence within *Azorina* at the end of Pleistocene which is similar with the divergence time estimated in this study of two *Azorina* subclades in the Azores archipelago ca. 0.15 Mya. The phylogenetic relationships and the estimation divergence times obtained in this study when compared with previous works determine the proximity of *A. vidalii*,* C. jacobaea* and *C. bravensis* with North African species, such as *C. edulis*,* C. mollis*,* C. kremeri*,* C. dichotoma*, and *C. occidentalis*, an endemic species from Canaries (García‐Aloy et al., 2017; Olesen et al., 2012; Roquet et al., 2008). How Macaronesia was colonized is yet uncertain, however several seamounts closer to Africa which were once islands, might have been stepping stones allowing many taxa to colonize the more distant islands (Fernández‐Palacios et al., 2011; Olesen et al., 2012), such as the Azores.

Olesen et al. (2012) assume that *A. vidalii* might first have colonized Santa Maria based in the divergence date obtained when compared with the age of the oldest island. However, we think that the beginning of the divergence of this species occurred before or during the dispersal events since the Africa to the Azores. Although the ages of the Azores islands are more recent than previously thought spanning from Santa Maria (4.1 Mya) to Pico (0.27 Mya) (Ávila et al., 2016; Sibrant, Hildenbrand, Marques, & Costa, 2015), our haplotype network indicates that the oldest haplotypes are in the central group, namely in São Jorge (1.32 Mya), which might have been first colonized by an *A. vidalii* ancestor.

Regarding the Cape Verde *Campanula*, Olesen et al. (2012) estimated that the ancestor of *C. jacobaea* colonized the islands in the recent past (1.4 Mya). Our results, which include for the first time *C. bravensis*, show a more recent time ca. 0.21 Mya. However, estimations of divergence times in Cape Verde are complex due to taxonomic uncertainty between *C. jacobaea* and *C. bravensis*.

A recent cladogenesis event appears to have occurred in the Macaronesian *C. erinus* (3.42 Mya), since the Pliocene.

The diversification of the living sisters of *Musschia* species such as *C. peregrina* Hoffm. & Link and *C. lactiflora* M. Bieb. distributed along the Mediterranean shores (Olesen et al., 2012) began at ca. 8.1 Mya. Olesen et al. (2012) indicated a separation between *M. aurea* and *M. wollastonii* in a process that started at 2.0 Mya. However, the past chronograms with divergence time estimations did not include *M. isambertoi*, an important species to understand the diversification within *Musschia*.

Our study shows that speciation of *Musschia* clade happened in the Pliocene (3.40 Mya), where an ancestral of *M. wollastonii* may have colonized Madeira, with a later cladogenesis resulting in the differentiation between *M. isambertoi* and *M. aurea* (0.07 Mya). Presently, *M. wollastonii* occurs in high altitudes inhabiting clearings of humid laurissilva forest, providing refuge sites for this species. Speciation into *M. isambertoi* and *M. aurea* resulted from adaptation to lower altitude coastal habitats. It is possible that *M. isambertoi* had a wider distribution and was later restricted to Deserta Grande due to extinction in Madeira Island. The estimated divergence times indicated a possible very recent separation between *M. aurea* (from Garajau population) and the putative *M. angustifolia* individuals. Other patterns of diversification according to the species habitat and the growth habit were also described for *Pericallis* D. Don in the Azores, Madeira, and Canaries which the origin of woodiness is correlated with ecological variation from open to species‐rich habitats and the ancestor of *Pericallis* was probably an herbaceous plant adapted to marginal habitats of the laurel forest (Panero, Francisco‐Ortega, Jansen, & Santos‐Guerra, 1999). Another pattern is reported for *Echium* L. which the islands colonization is related to the origin of perennial woodiness from herbaceous habit and was furthermore accompanied by intense speciation (Böhle, Hilger, & Martin, 1996).

In what concern to the Macaronesian endemic *W. lobelioides* subsp. *lobelioides*, it seems that colonization initiated in Canaries and later dispersed to the other archipelagos, dated 0.87 Mya, being possibly the oldest lineage in this biogeographic region. This species could have colonized first Cape Verde (0.07 Mya), and then Madeira and Porto Santo (0.04 Mya). However, repeated events of colonization between Madeira and Cape Verde might explain the distinct clade within *W. lobelioides* subsp. *lobelioides* dated ca. 0.01 Mya.

### 4.3 Conservation approaches

The plant conservation strategy in Macaronesia has a clear focus on threatened endemic taxa, but only some of the study species were assessed and included in the IUCN Red List of Threatened Species (www.iucnredlist.org). As suggested by Romeiras, Catarino, Filipe, et al. (2016), new prioritization methods should consider the spatial and intra‐archipelago genetic diversity of insular taxa. Due to their uncertain status, most of this study target taxa are not currently protected, with some exceptions. *Musschia isambertoi* is the rarest species of *Musschia* and was proposed the IUCN category “Critically Endangered” (CR,C2a(i,ii);D) by Sequeira et al. (2007), although included in a protected area (Natura 2000 EU PTDES0001) the recent uncontrolled increase of population numbers of the common goat drove this taxon to the edge of extinction. Moreover, Romeiras, Catarino, Gomes, et al. (2016) proposed the IUCN category of “Endangered” (B1ab(ii,iv)+2ab(ii,iv)) for *C. bravensis* and “Vulnerable” (B1ab(ii)+2ab(ii)) for *C. jacobaea*, and confirmed that the Cape Verde vascular plants have become more threatened and their conservation status has declined in the last years, mostly as a consequence of the increase in exotic species, habitat degradation, and human disturbance. Conservations plans are needed in order to preserve the Cape Verdean Campanulaceae (see for details Romeiras, Catarino, Gomes, et al., 2016).

The genetic diversity found within the studied taxa among the archipelagos and the several single‐island haplotypes observed must be considered and protected. Although there is a clear lack of taxonomic revisions including insular taxa usually considered as native, further studies in population genetics structure and reproductive biology should also be conducted, as proposed by Silva et al. (2015) in a holistic approach to conservation of rare island plants. Furthermore, to define better conservation strategies of the Macaronesian endemic flora, prioritizing threatened species and conserving the entire extent of their natural ranges was recently recognized as a crucial step (Romeiras, Catarino, Gomes, et al., 2016; Romeiras, Vieira, et al., 2016).

Concerning the colonization status of the target Campanulaceae, as described in the regional checklists (Ginovés et al., 2010; Menezes de Sequeira et al., 2012; Sánchez‐Pinto et al., 2005; Silva et al., 2010), the phylogenetic data generated in this study provides an additional knowledge to understand the doubtful status about the colonization of these taxa: (1) *C. erinus* status of “naturalized” for the Azores (Silva et al., 2010) and “doubtfully native” for the Canary Islands (Ginovés et al., 2010) should be changed to “native”; (2) *T. caeruleum*, listed as “doubtfully naturalized” for the Azores (Silva et al., 2010) needs an in‐depth study to assert a native status; and (3) *W. lobelioides* subsp. *lobelioides*, listed “doubtfully native” for Cape Verde, should be considered native.

This study shows that the spatial patterns of species differ among the studied Campanulaceae lineages as well as their evolutionary history within the Macaronesian archipelagos. Hence, future conservation measures should consider the existing inter and intra‐archipelago genetic variation in the Campanulaceae family in Macaronesia, and accordingly, priority taxa should not be restricted to endemic lineages, but also include native threatened species.

## AUTHOR'S CONTRIBUTIONS

M.M., M.M.R., and T.M. contributed to design the study. T.M., M.M.R., and M.M.S. did the sampling. T.M. did the laboratory work and ran the molecular data analyses; T.M. and M.M. analyzed and interpreted the results; T.M. led the writing with substantial contributions from all co‐authors. All authors approved the final article.
