# Supplementary material for: Phylogenetic relationships and phylogeography of relevant lineages within the complex Campanulaceae family in Macaronesia
Source: Ecol Evol. 2017 Nov 23;8(1):88–108. doi: 10.1002/ece3.3640 (PMC5756848; doi:10.1002/ece3.3640)
Supplement: Supplementary file 1 [file ECE3-8-88-s001.docx]

**SUPPORTING INFORMATION**

**Supplementary Table 1.** List of all Campanulaceae sequences data available on GenBank (<http://www.ncbi.nlm.nih.gov/genbank>) used in this study.

| Species | ITS | *matK* | *rbcL* | *trnL-F* | *petD* |
| --- | --- | --- | --- | --- | --- |
| *Campanula alliariifolia* | AY322008 | EU713269 | EU713376 | EF088700 | JX915112 |
| *Campanula arvatica* | AY322010 | EU713344 | EU713451 | - | JX915113 |
| *Campanula aucheri* | EF090547 | EU713241 | EU713348 | - | JX914754 |
| *Campanula barbata* | AY322011 | KJ512559 | KJ512614 | KJ512658 | JX914740 |
| *Campanula bellidifolia* | AY322012 | EU713240 | EU713347 | EF088706 | JX915115 |
| *Campanula bononiensis* | DQ304571 | EU713274 | EU713381 | KJ512663 | JX914730 |
| *Campanula caespitosa* | DQ304621 | KJ512567 | KJ512624 | KJ512655 | JX915155 |
| *Campanula carpatha* | - | EU713261 | EU713368 | KJ512672 | JX915209 |
| *Campanula carpatica* | AY322013 | EU713303 | EU713410 | - | JX915231 |
| *Campanula cenisia* | - | KJ512570 | KJ512627 | KJ512673 | JX915211 |
| *Campanula creutzburgii* | - | EU713290 | EU713397 | EF088714 | JX915210 |
| *Campanula divaricata* | AY322014 | EU713343 | EU713450 | EF088718 | FN397005 |
| *Campanula drabifolia* | DQ304578 | KJ512556 | KJ512629 | EF088719 | JX915174 |
| *Campanula edulis* | AY322015 | EU713267 | EU713374 | JF747590 | JX915236 |
| *Campanula elatines* | DQ304624 | EU713331 | EU713438 | FJ426577 | JX915116 |
| *Campanula elatinoides* | DQ304625 | KJ512572 | KJ512630 | FJ426578 | FN397008 |
| *Campanula erinus* | DQ304580* | EU713291 | EU713398 | EF088720 | JX915158 |
| *Campanula fragilis* | DQ304627 | EU713321 | EU713428 | FJ426580 | JX915237 |
| *Campanula glomerata* | AY322017 | KJ512576 | KJ512634 | KJ512678 | JX914719 |
| *Campanula grossheimii* | AY322018 | EU713286 | EU713393 | - | JX915118 |
| *Campanula hawkinsiana* | AY322019 | EU713338 | EU713445 | EF213146 | JX915186 |
| *Campanula herminii* | AY322020 | EU713340 | EU713447 | JF747588 | JX915120 |
| *Campanula lactiflora* | EU177776 | KJ512577 | KJ512635 | - | JX915131 |
| *Campanula lanata* | AY322023 | EU713275 | EU713382 | - | JX914953 |
| *Campanula latifolia* | AY322024 | EU713271 | EU713378 | - | JX914791 |
| *Campanula lusitanica* | HQ407553 | EU713334 | EU713441 | EF088733 | JX915122 |
| *Campanula marchesettii* | DQ304612 | KJ512580 | KJ512638 | KJ512685 | JX915167 |
| *Campanula medium* | EF090537 | EU713272 | EU713379 | - | FN397024 |
| *Campanula mirabilis* | AY322026 | EU713277 | EU713384 | - | JX915123 |
| *Campanula mollis* | AY322027 | EU713268 | EU713375 | KC792346 | JX915124 |
| *Campanula parryi* | JN571994 | EU713342 | EU713449 | EF213147 | KJ512603 |
| *Campanula patula* | FM212739 | KJ512582 | KJ512640 | EF213148 | JX914974 |
| *Campanula peregrina* | EU177772 | EU713320 | EU713427 | EF088742 | JX915125 |
| *Campanula persicifolia* | DQ304590 | EU713324 | EU713431 | EF213149 | JX915126 |
| *Campanula petraea* | AY331444 | KJ512583 | KJ512641 | KJ512688 | JX915207 |
| *Campanula portenschlagiana* | DQ304600 | KJ512584 | KJ512642 | FJ426587 | JX915190 |
| *Campanula pyramidalis* | DQ304606 | EU713322 | EU713429 | GQ254919 | JX915192 |

(*Continued*)

**Supplementary Table 1.** (*Continued*)

| Species | ITS | *matK* | *rbcL* | *TrnL-F* | *petD* |
| --- | --- | --- | --- | --- | --- |
| *Campanula rapunculoides* | EF090545 | EU713285 | EU713392 | EF213152 | JX915191 |
| *Campanula rapunculus* | FM212738 | KJ512585 | KJ512643 | - | JX914708 |
| *Campanula rotundifolia* | JN571988 | EU713335 | EU713442 | EF213153 | JX915229 |
| *Campanula sarmatica* | AY322038 | EU713279 | EU713386 | GQ254921 | JX915130 |
| *Campanula scheuchzeri* | DQ304614 | KJ512588 | KJ512646 | - | JX915162 |
| *Campanula sibirica* | EF090550 | KJ512589 | KJ512647 | EF213157 | JX914681 |
| *Campanula spicata* | DQ304574 | KJ512591 | KJ512649 | EF088769 | FN397044 |
| *Campanula stenocodon* | DQ304620 | KJ512592 | KJ512650 | KJ512697 | KJ512604 |
| *Campanula thyrsoides* | AY331455 | KJ512593 | KJ512651 | KJ512699 | FN397046 |
| *Campanula versicolor* | DQ304607 | KJ512594 | KJ512652 | FJ426591 | JX915228 |
| *Canarina canariensis* | AY322045 | EU713246 | EU713353 | - | FN397055 |
| *Edraianthus graminifolius* | AY322052 | KJ512596 | KJ512654 | - | JX914929 |
| *Feeria angustifolia* | AY322054 | EU713287 | EU713394 | - | JX914795 |
| *Githopsis diffusa* | AY322056 | EU713310 | EU713417 | - | JX915141 |
| *Hanabusaya asiatica* | HQ704540 | EU713325 | EU713432 | - | JX915148 |
| *Heterocodon rariflorum* | AY322058 | EU713307 | EU713414 | - | JX915149 |
| *Jasione crispa* | DQ304567 | EU713283 | EU713390 | - | JX915150 |
| *Jasione heldreichii* | DQ222834 | EU713281 | EU713388 | - | JX915151 |
| *Jasione laevis* | DQ222845 | EU713280 | EU713387 | - | FN397069 |
| *Jasione montana* | DQ304566 | EU713247 | EU713354 | - | JX915216 |
| *Legousia falcata* | DQ304589 | EU713311 | EU713418 | - | JX915153 |
| *Legousia hybrida* | EF090558 | EU713327 | EU713434 | - | - |
| *Legousia speculum* | AY331478 | EU713258 | EU713365 | KJ512701 | FN397071 |
| *Michauxia tchihatcheffii* | AY322068 | EU713239 | EU713346 | - | JX914981 |
| *Petromarula pinnata* | DQ304582 | EU713326 | EU713433 | FJ426585 | JX915212 |
| *Physoplexis comosa* | DQ304585 | EU713255 | EU713362 | - | JX915240 |
| *Platycodon grandiflorus* | AY331486 | EU713251 | EU713358 | EF088788 | FN397087 |
| *Trachelium caeruleum* | DQ304569* | EU713328* | EU713435* | - | JX914943* |
| *Wahlenbergia hederacea* | HQ407556 | EU713293 | EU713400 | EF088792 | JX915241 |
| *Cyphia decora* | - | - | - | GQ984060 | - |
| *Cyphia elata* | - | EU713264 | EU713371 | - | - |
| *Cyphia tysonii* | - | - | - | - | FN397062 |
| *Cyphia comptonii* | KC013706 | - | - | - | - |
| *Cyphia rogersii* | - | - | - | - | - |

*the sequence was used to generate the haplotype networks.

**Supplementary Table 2.** Description of Campanulaceae vouchers and GenBank accession numbers for the corresponding ITS and cpDNA sequences (<http://www.ncbi.nlm.nih.gov/genbank>). For the samples collected for this study, the following data are provided: sampling sites, vouchers and geographic coordinates.

| **Taxon** | **Sampling locations** | **GenBank accession numbers** | | | | | | | **Herbarium and codes** | | **Geographic coordinates** | |
| --- | --- | --- | --- | --- | --- | --- | --- | --- | --- | --- | --- | --- |
|  |  | **ITS** | ***matK*** | ***rbcL*** | ***psbA-trnH*** | ***trnL-F*** | ***petD*** | ***atpB*** |  |  | **Latitude** | **Longitude** |
| *Azorina vidalii* | |  |  |  |  |  |  |  |  |  |  |  |
|  | Azores, São Miguel, São Vicente | KY091344* | KY091392 | KY091530 | KY091490 | KY091575 | KY091444 | KY091318 | AZB | AV-SMSV-04 | 35,835000 | -25,673056 |
|  | Azores, São Miguel, Mosteiros | KY091343* | KY091391 | KY091529 | KY091489 | KY091574 | KY091443 | KY091317 | AZB | AV-SMMO-06 | 37,898611 | -25,821667 |
|  | Azores, Santa Maria, Ponta do Castelo | KY091340* | KY091388 | KY091526 | KY091486 | KY091571 | KY091440 | KY091314 | AZB | AV-MAPC-01 | 36,928611 | -25,016944 |
|  | Azores, Terceira, Porto Martins | KY091346* | KY091394 | KY091532 | KY091492 | KY091577 | KY091446 | KY091320 | AZB | AV-TEPM-01 | 38,684167 | -27,057222 |
|  | Azores, Terceira, Porto Judeu | KY091345* | KY091393 | KY091531 | KY091491 | KY091576 | KY091445 | KY091319 | AZB | AV-TEPJ-04 | 38,646441 | -27,122406 |
|  | Azores, Faial, Jardim Botânico | KY091339* | KY091387 | KY091525 | KY091485 | KY091570 | KY091439 | KY091313 | AZB | AV-FAJB-02 | 38,550833 | -28,639167 |
|  | Azores, Pico, Santo Amaro | KY091341* | KY091389 | KY091527 | KY091487 | KY091572 | KY091441 | KY091315 | AZB | AV-PIAM-02 | 38,457778 | -28,173333 |
|  | Azores, Pico, Baixa da Ribeirinha | KY091342* | KY091390 | KY091528 | KY091488 | KY091573 | KY091442 | KY091316 | AZB | AV-PIBR-05 | 38,444722 | -28.077500 |
| *Campanula bravensis* | |  |  |  |  |  |  |  |  |  |  |  |
|  | Cape Verde, Fogo, Ribeira do Coxo | KY091379* | KY091425 | KY091557 | KY091514 | KY091608 | KY091447 | - | LISC | 760 | 15,018056 | -24,397222 |
|  | Cape Verde, Fogo, Bordeira, Gruta Cruz | KY091380* | KY091426 | KY091558 | KY091515 | KY091609 | KY091448 | - | LISC | 762 | 14,914408 | -24,351089 |
|  | Cape Verde, Brava, Cruz da Fajã | - | KY091427 | KY091559 | KY091516 | KY091610 | KY091449 | - | LISC | 537 | 14,872933 | -24,725117 |
|  | Cape Verde, Brava, Cruz da Fajã | - | KY091428 | KY091560 | KY091517 | KY091611 | KY091450 | - | LISC | 5816 | 14,872933 | -24,725117 |
| *Campanula erinus* | |  |  |  |  |  |  |  |  |  |  |  |
|  | Azores, Santa Maria, Praia Formosa | KY091348* | KY091395 | KY091534 | KY091494 | KY091579 | - | - | AZU | 5084 | - | - |
|  | Azores, São Miguel, Paím | KY091358* | KY091405 | - | - | - | KY091456 | - | AZB | CE-SMIP-01 | 37,748074 | -25,685499 |
|  | Azores, São Miguel, Conceição | KY091357* | KY091404 | KY091539 | - | KY091588 | KY091457 | - | AZB | CE-SMCO-01 | 37,817831 | -25,529909 |
|  | Madeira, Madeira Island, Porto Novo | KY091354* | KY091401 | KY091536 | KY091498 | KY091585 | KY091453 | - | AZB | CE-MDPN-01 | 32,661718 | -16,813637 |
|  | Madeira, Porto Santo, Rocha de Nª. Senhora | KY091355* | KY091402 | KY091537 | KY091499 | KY091586 | KY091454 | - | AZB | CE-PSRS-01 | 33,073912 | -16,323809 |
|  | Madeira, Porto Santo, Rocha de Nª. Senhora | KY091356* | KY091403 | KY091538 | KY091500 | KY091587 | KY091455 | - | AZB | CE-PSRS-02 | 33,073912 | -16,323809 |
|  | Madeira, Madeira Island, Curral das Freiras | KY091353* | KY091400 | KY091535 | KY091497 | KY091584 | KY091452 | - | AZB | CE-MDCF-01 | 32,726697 | -16,966312 |
|  | Canary Islands, Tenerife, Masca | KY091349* | KY091396 | - | KY091495 | KY091580 | - | - | ORT | 13035 | - | - |
|  | Canary Islands, Tenerife, Los Silos | KY091350* | KY091397 | - | KY091496 | KY091581 | - | - | ORT | 13039 | - | - |
|  | Canary Islands, La Palma, Sta. Cecilia | KY091347* | - | KY091533 | KY091493 | KY091578 | - | - | ORT | 3462 | - | - |
|  | Canary Islands, La Palma, Tijarate | KY091352* | KY091399 | - | - | KY091583 | - | - | ORT | 30583 | - | - |
|  | Canary Islands, El Hierro, El Golfo | KY091351* | KY091398 | - | - | KY091582 | KY091451 | - | ORT | 17168 | - | - |
| *Campanula jacobaea* | |  |  |  |  |  |  |  |  |  |  |  |
|  | Cape Verde, São Nicolau, Pico da Cruz | KY091386* | KY091434 | KY091566 | KY091523 | KY091617 | KY091463 | - | LISC | 4078 | 17,099975 | -25,020169 |
|  | Cape Verde, São Nicolau, Monte Gordo | - | KY091435 | KY111276 | KY091524 | KY091618 | KY091464 | - | LISC | 4128 | 16,627783 | -24,354167 |
|  | Cape Verde, Santiago, Serra da Malagueta | KY091382* | KY091430 | KY091562 | KY091519 | KY091613 | KY091459 | - | LISC | 1001 | 15,178450 | -24,397133 |
|  | Cape Verde, Santiago, Serra da Malagueta | KY091381* | KY091429 | KY091561 | KY091518 | KY091612 | KY091458 | - | LISC | 1000 | 15,178450 | -24,397133 |
|  | Cape Verde, São Vicente, Monte Verde | KY091385* | KY091433 | KY091565 | KY091522 | KY091616 | KY091462 | KY091322 | LISC | 3219 | 16,870744 | -24,933681 |
|  | Cape Verde, Santo Antão, Cova | KY091383* | KY091431 | KY091563 | KY091520 | KY091614 | KY091460 | - | LISC | 1076 | 17,104244 | -25,060806 |
|  | Cape Verde, Santo Antão, Maroços | KY091384* | KY091432 | KY091564 | KY091521 | KY091615 | KY091461 | KY091321 | LISC | 1095 | 17,081389 | -25,145083 |
| *Lobelia urens* | |  |  |  |  |  |  |  |  |  |  |  |
|  | Madeira, Madeira Island, Ginjas | KY091360 | KY091407 | - | KY091502 | KY091590 | KY091466 | - | AZB | LU-MDGL-02 | 32,771668 | -17,055302 |
|  | Madeira, Madeira Island, Fanal | KY091359 | KY091406 | - | KY091501 | KY091589 | KY091465 | - | AZB | LU-MDFA-01 | 32,826392 | -17,158052 |

(*Continued*)

**Supplementary Table 2.** (*Continued*)

| **Taxon** | **Sampling locations** | **GenBank accession numbers** | | | | | | | **Herbarium and codes** | | **Geographic coordinates** | |
| --- | --- | --- | --- | --- | --- | --- | --- | --- | --- | --- | --- | --- |
|  |  | **ITS** | ***matK*** | ***rbcL*** | ***psbA-trnH*** | ***trnL-F*** | ***petD*** | ***atpB*** |  |  | **Latitude** | **Longitude** |
| *Musschia aurea* | |  |  |  |  |  |  |  |  |  |  |  |
|  | Madeira, Madeira Island, Garajau | KY091365* | KY091413* | KY091545* | KY091507 | KY091596* | KY091471 | KY091327 | AZB | MU-MDGA-07 | 32,640640 | -16,848953 |
|  | Madeira, Madeira Island, Garajau | KY091366* | KY091414* | KY091546* | KY091508 | KY091597* | KY091472 | KY091328 | AZB | MU-MDGA-18 | 32,639859 | -16,852935 |
|  | Madeira, Desertas, Deserta Grande | KY091363* | KY091411* | KY091543* | KY091505 | KY091594* | KY091469 | KY091325 | AZB | MU-DEDG-01 | 32,513551 | -16,505542 |
|  | Madeira, Desertas, Deserta Grande | KY091364* | KY091412* | KY091544* | KY091506 | KY091595* | KY091470 | KY091326 | AZB | MU-DEDG-02 | 32,513407 | -16,506228 |
|  | Madeira, Madeira Island, Ponta Delgada | KY091362* | KY091409* | KY091541* | KY091504 | KY091592* | KY091468 | KY091324 | AZB | MF-MDPD-01 | - | - |
|  | Madeira, Madeira Island, Porto da Cruz | KY091361* | KY091408* | KY091540* | KY091503 | KY091591* | KY091467 | KY091323 | AZB | MF-MDCM-03 | 32,765115 | -16,822029 |
| *Musschia isambertoi* | |  |  |  |  |  |  |  |  |  |  |  |
|  | Madeira, Desertas, Deserta Grande | KY091367* | KY091410* | KY091542* | - | KY091593* | - | KY091330 | AZB | MI-DEDG-01 | 32,514200 | -16,507081 |
| *Musschia wollastonii* | |  |  |  |  |  |  |  |  |  |  |  |
|  | Madeira, Madeira Island, Levada do Folhadal | KY091368* | KY091415* | KY091547* | KY091509 | KY091598* | KY091473 | - | AZB | MW-MDET-05 | 32,751893 | -17,040269 |
|  | Madeira, Madeira Island, Levada do Folhadal | KY091369* | KY091416* | KY091548* | KY091510 | KY091599* | KY091474 | KY091329 | AZB | MW-MDET-21 | 32,752529 | -17,041060 |
| *Trachelium caeruleum* | |  |  |  |  |  |  |  |  |  |  |  |
|  | Madeira, Madeira Island, Funchal, Alegria | KY091370* | KY091417* | KY091549* | KY091511 | KY091600 | KY091475* | KY091331 | AZB | TC-MDFU-01 | 32,684943 | -16,926108 |
|  | Azores, São Miguel, Água de Pau | KY091371* | KY091418* | KY091550* | KY091512 | KY091601 | KY091476* | KY091332 | AZB | TC-SMAP-01 | 37,722778 | -25,516667 |
|  | Azores, São Miguel, Porto Formoso | KY091372* | KY091419* | KY091551* | KY091513 | KY091602 | KY091477* | KY091333 | AZB | TC-SMFO-01 | 37,816389 | -25.422500 |
| *Wahlenbergia lobelioides* subsp. *lobelioides* | |  |  |  |  |  |  |  |  |  |  |  |
|  | Madeira, Porto Santo, Ilhéu de Cima | KY091375* | KY091423 | KY091555 | - | KY091606 | KY091483 | KY091337 | AZB | WL-PSCI-01 | 33,053166 | -16,279019 |
|  | Madeira, Madeira Island, Ribeira Brava | KY091378* | KY091422 | KY091554 | - | KY091605 | KY091482 | KY091338 | AZB | WL-MDRB-02 | 32,663864 | -17,037735 |
|  | Madeira, Porto Santo, Pico Facho | KY091376* | KY091424 | KY091556 | - | KY091607 | KY091484 | - | AZB | WL-PSPF-03 | 33,084087 | -16,326308 |
|  | Madeira, Madeira Island, Paúl do Mar | KY091377* | KY091421 | KY091553 | - | KY091604 | KY091481 | - | AZB | WL-MDPL-01 | 32,754345 | -17,223902 |
|  | Canary Islands, Lanzarote, Haria Guimate | KY091374* | KY091420 | KY091552 | - | KY091603 | - | - | ORT | 41666 | - | - |
|  | Cape Verde, São Nicolau, Monte Gordo | KY091373* | KY091436 | KY091567 | - | KY091619 | KY091478 | KY091334 | LISC | 2906 | - | - |
|  | Cape Verde, Fogo, Chã de Ribeira | - | KY091437 | KY091568 | - | KY091620 | KY091479 | KY091335 | LISC | 3597 | - | - |
|  | Cape Verde, Santo Antão, Ribeira do Paúl | - | KY091438 | KY091569 | - | KY091621 | KY091480 | KY091336 | LISC | 7068 | - | - |

*the sequence was used to generate the haplotype networks.

To generate the haplotype network for *Azorina vidalii* were used the ITS sequences from Schaefer *et al*. (2011) available on supporting information section of Journal of Biogeography on website (<http://onlinelibrary.wiley.com/doi/10.1111/j.1365-2699.2011.02494.x/full>).

**Supplementary Table 3.** Parameters of the best parsimonious tree for the markers tested.

| **Markers tested** | **Number of samples** | **Alignment length (base pairs)** | **Informative characters** | **Length** | **CI*** | **RI**** | **HI***** |
| --- | --- | --- | --- | --- | --- | --- | --- |
| **ITS** | 111 | 928 | 419 | 2133 | 0,44 | 0,81 | 0,56 |
| ***matK*** | 118 | 895 | 311 | 835 | 0,69 | 0,92 | 0,31 |
| ***rbcL*** | 112 | 714 | 101 | 460 | 0,49 | 0,70 | 0,52 |
| ***psbA-trnH*** | 40 | 438 | - | - | - | - | - |
| ***trnL-F*** | 84 | 510 | 176 | 717 | 0,78 | 0,85 | 0,23 |
| ***pet-D*** | 11 | 1008 | 297 | 785 | 0,71 | 0,92 | 0,29 |
| ***atpB*** | 29 | 917 | 98 | 378 | 0,97 | 0,98 | 0,03 |
| **Concatenate cp markers** | 119 | 4482 | 1485 | 5151 | 0,69 | 0,86 | 0,31 |
| **Concatenate ITS + cp markers** | 119 | 5410 | 1904 | 7005 | 0,64 | 0,88 | 0,37 |

* CI, Consistency index value; ** RI, Retention index value; *** HI, Homoplasy index value.

**
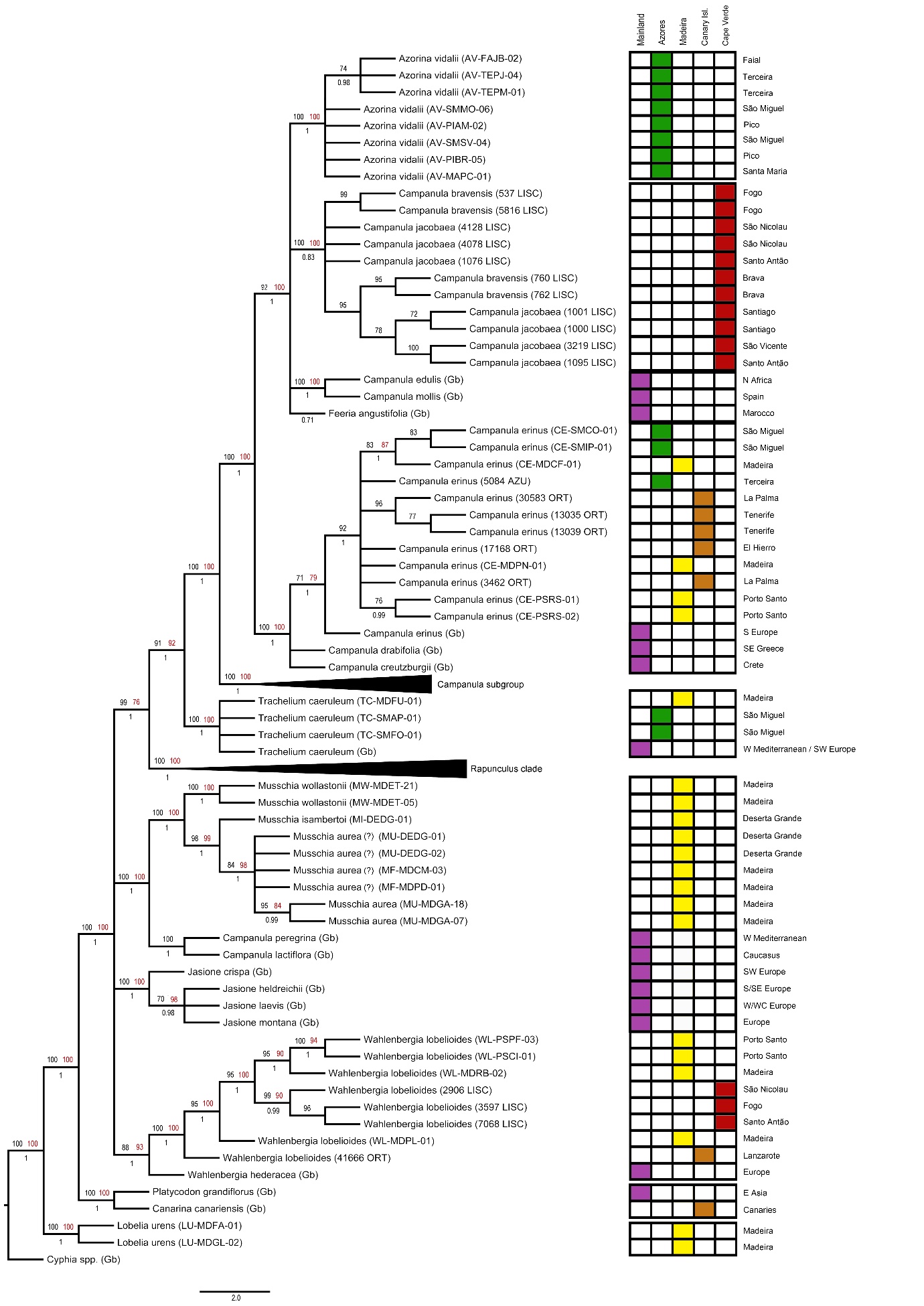
Figure S1.** Combined Plastid and ITS Phylogeny and species distributions. Best tree from maximum likelihood analysis of concatenated plastid-ITS dataset. Numbers above branches (≥70%) are: black) maximum likelihood; red) maximum parsimony bootstrap values; Number bellow branches (≥0,70) are bayesian posterior probabilities. Sequences of taxa labelled with “(Gb)” were obtained on GenBank (Supplementary Table 2).


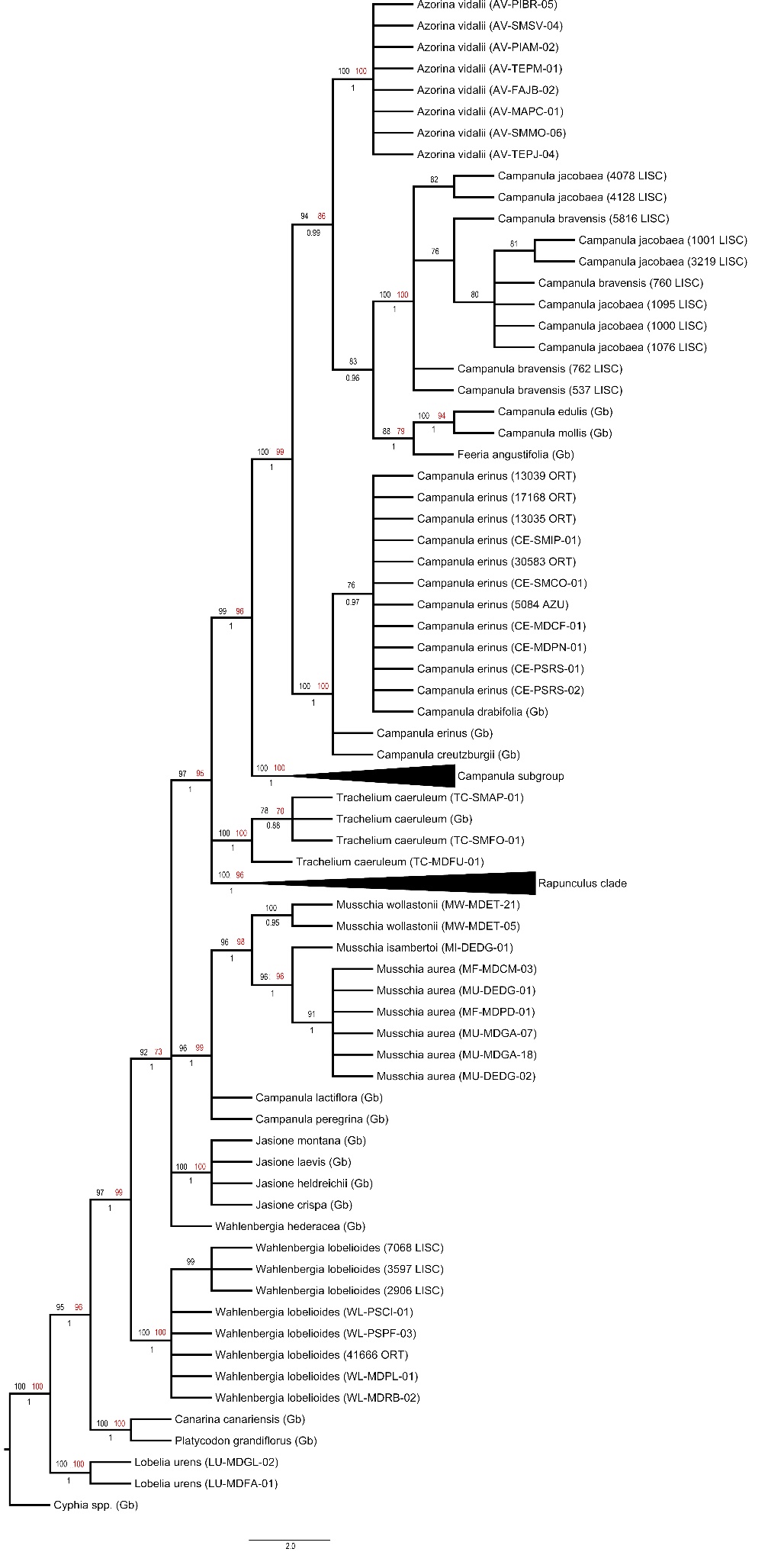


**Figure S2.** *matK* Phylogeny. Best tree from maximum likelihood analysis. Numbers above branches (≥70%) are: black) maximum likelihood; red) maximum parsimony bootstrap values; Number bellow branches (≥0,70) are bayesian posterior probabilities. Sequences of taxa labelled with “(Gb)” were obtained on GenBank (Supplementary Table 2).


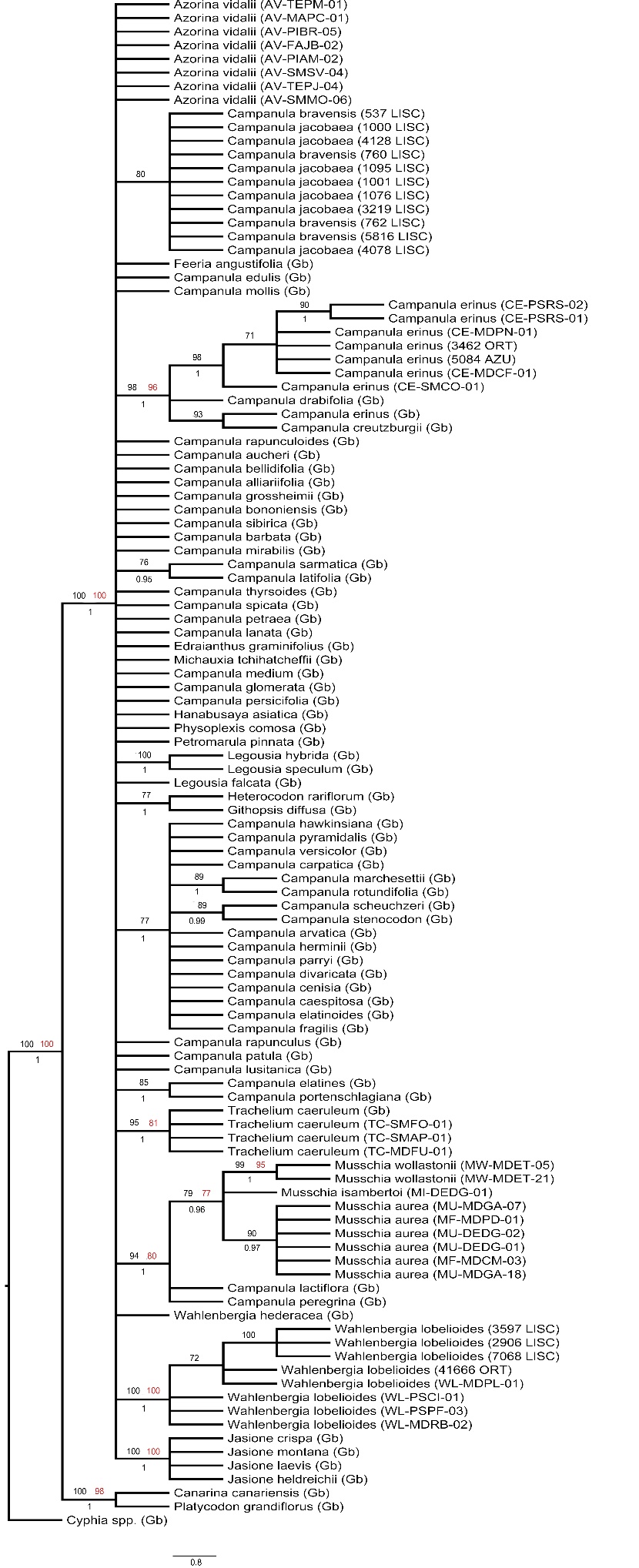


**Figure S3.** *rbcL* Phylogeny. Best tree from maximum likelihood analysis. Numbers above branches (≥70%) are: black) maximum likelihood; red) maximum parsimony bootstrap values; Number bellow branches (≥0,70) are bayesian posterior probabilities. Sequences of taxa labelled with “(Gb)” were obtained on GenBank (Supplementary Table 2).


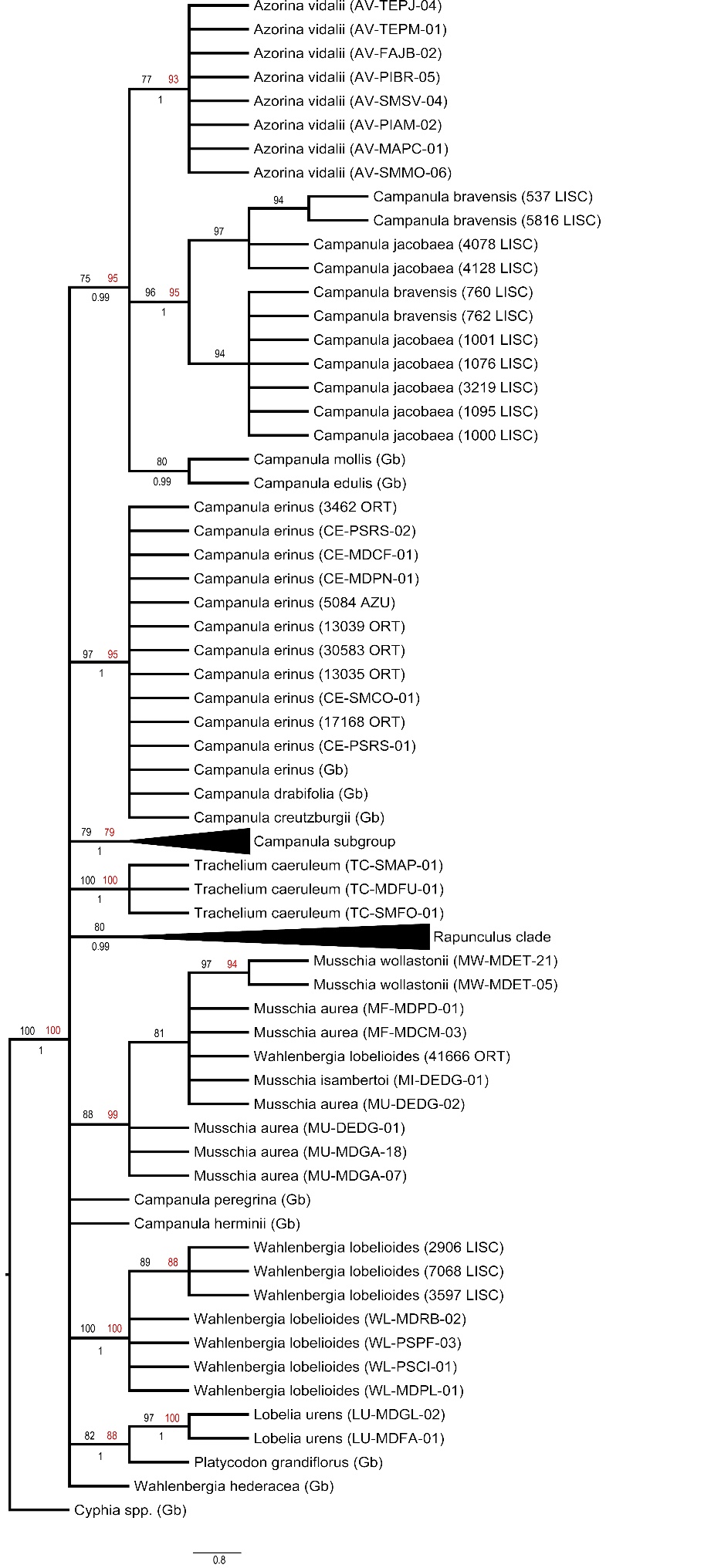


**Figure S4.** *trnL-F* Phylogeny. Best tree from maximum likelihood analysis. Numbers above branches (≥70%) are: black) maximum likelihood; red) maximum parsimony bootstrap values; Number bellow branches (≥0,70) are bayesian posterior probabilities. Sequences of taxa labelled with “(Gb)” were obtained on GenBank (Supplementary Table 2).


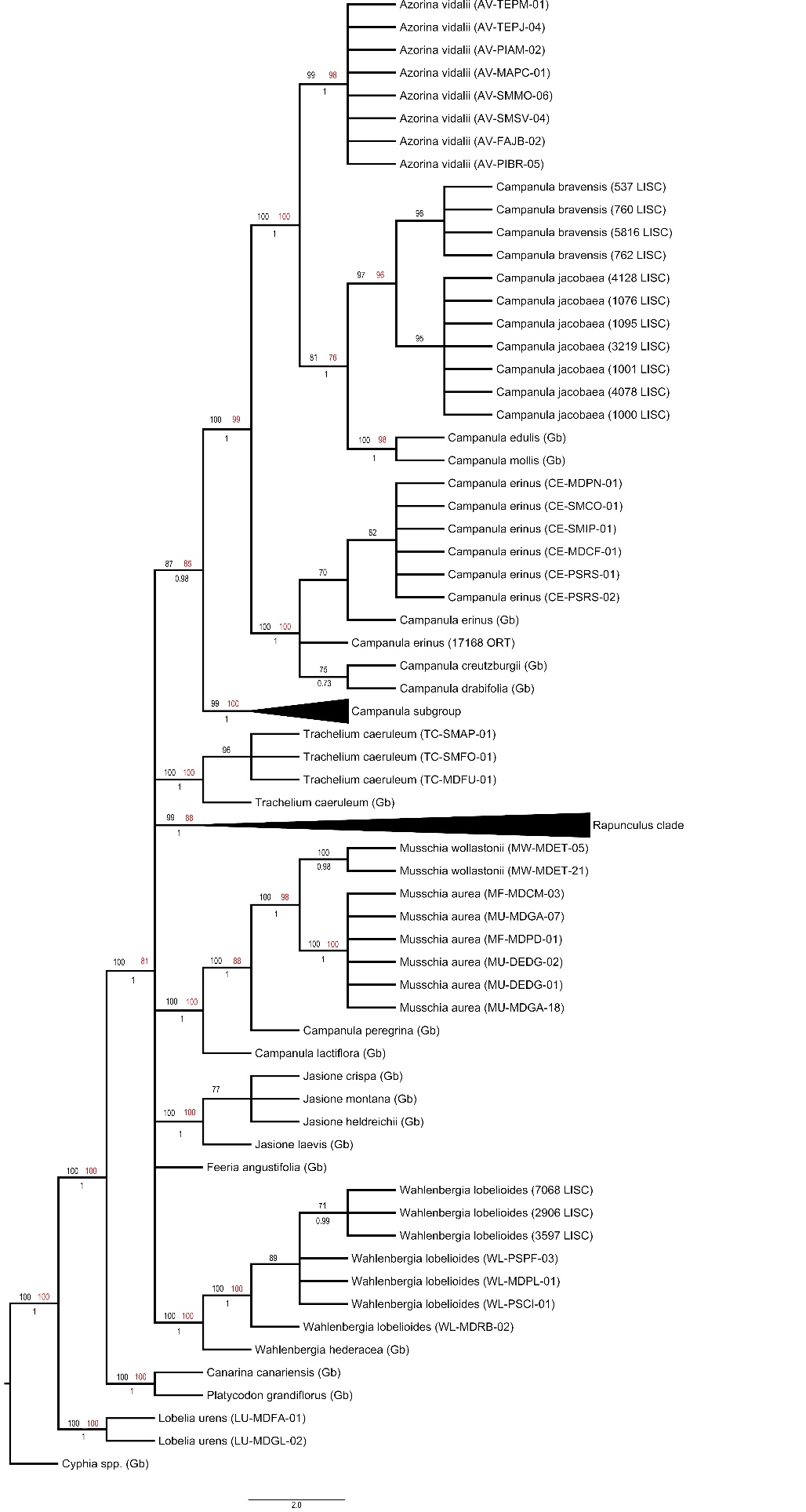


**Figure S5.** *pet-D* Phylogeny. Best tree from maximum likelihood analysis. Numbers above branches (≥70%) are: black) maximum likelihood; red) maximum parsimony bootstrap values; Number bellow branches (≥0,70) are bayesian posterior probabilities. Sequences of taxa labelled with “(Gb)” were obtained on GenBank (Supplementary Table 2).


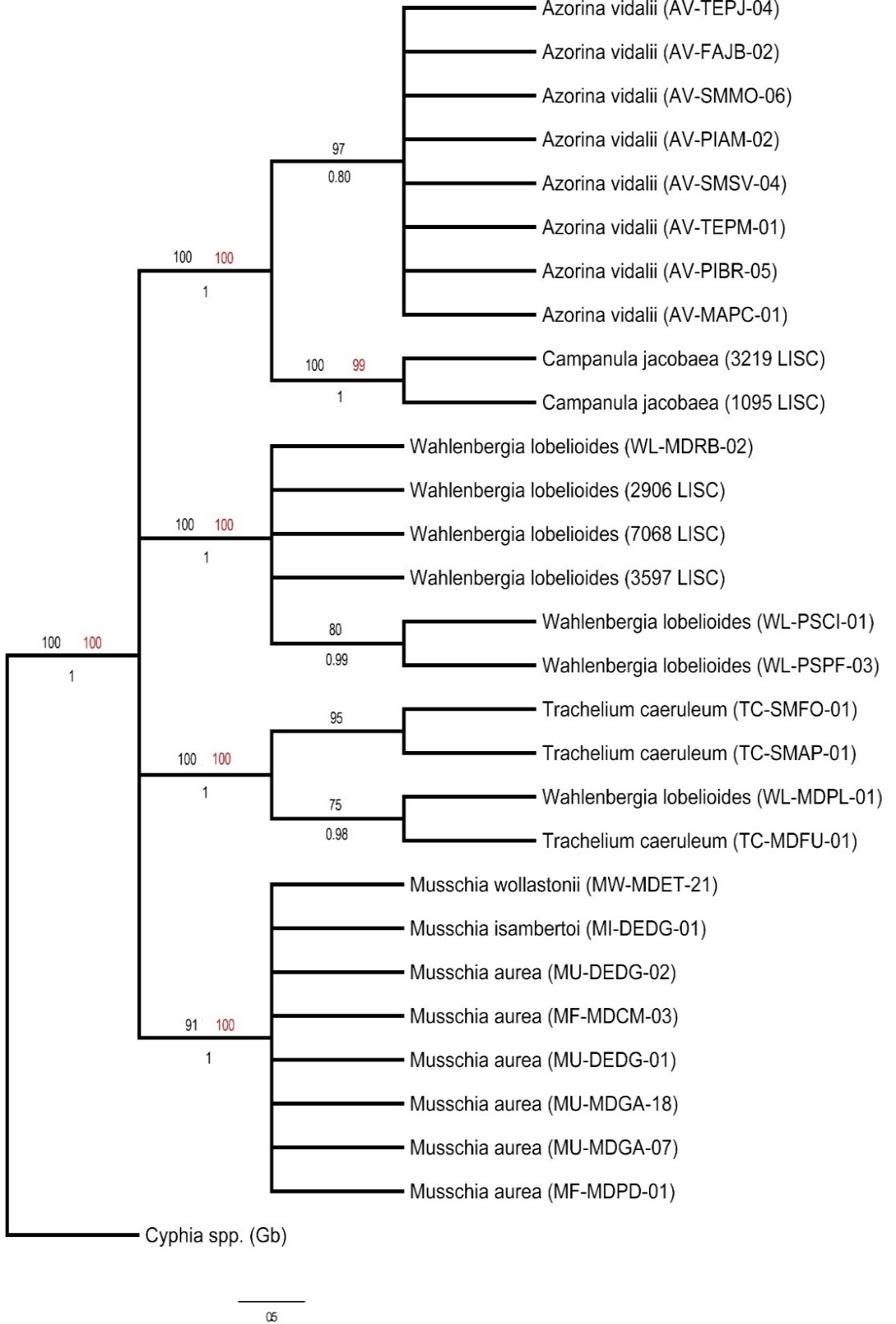


**Figure S6.** *atpB* Phylogeny. Best tree from maximum likelihood analysis. Numbers above branches (≥70%) are: black) maximum likelihood; red) maximum parsimony bootstrap values; Number bellow branches (≥0,70) are bayesian posterior probabilities. Sequences of taxa labelled with “(Gb)” were obtained on GenBank (Supplementary Table 2).
